# BM-MSCs display altered gene expression profiles in B-cell acute lymphoblastic leukemia niches and exert pro-proliferative effects via overexpression of IFI6

**DOI:** 10.1186/s12967-023-04464-1

**Published:** 2023-09-05

**Authors:** Chengyun Pan, Tianzhen Hu, Ping Liu, Dan Ma, Shuyun Cao, Qin Shang, Luxin Zhang, Qingzhen Chen, Qin Fang, Jishi Wang

**Affiliations:** 1 Department of Hematology, Affiliated Hospital of Guizhou Medical University, 28 Guiyi St., Yunyan District, Guiyang, 550004 Guizhou China; 2 School of Basic Medical Sciences, Guizhou Medical University, Guizhou, China; 3 Hematological Institute of Guizhou Province, Guizhou, China; 4 Guizhou Province Hematopoietic Stem Cell Transplantation Centre and Key Laboratory of Hematological Disease Diagnostic and Treatment Centre, Guizhou, China; 5 Department of Pharmacy, Affiliated Hospital of Guizhou Medical University, 28 Guiyi St., Yunyan District, Guiyang, 550004 Guizhou China

**Keywords:** B-cell acute lymphoblastic leukemia, Tumor microenvironment, Mesenchymal stem cells, Interferon alpha-inducible protein 6, Proliferation

## Abstract

**Background:**

The tumor microenvironment (TME) is a supportive environment responsible for promoting the growth and proliferation of tumor cells. Current studies have revealed that the bone marrow mesenchymal stem cells (BM-MSCs), a type of crucial stromal cells in the TME, can promote the malignant progression of tumors. However, in the adult B-cell acute lymphoblastic leukemia (B-ALL) microenvironment, it is still uncertain what changes in BM-MSCs are induced by leukemia cells.

**Methods:**

In this study, we mimicked the leukemia microenvironment by constructing a BM-MSC–leukemia cell co-culture system. In vitro cell experiments, in vivo mouse model experiments, lentiviral transfection and transcriptome sequencing analysis were used to investigate the possible change of BM-MSCs in the leukemia niche and the potential factors in BM-MSCs that promote the progression of leukemia.

**Results:**

In the leukemia niche, the leukemia cells reduced the MSCs' capacity to differentiate towards adipogenic and osteogenic subtypes, which also promoted the senescence and cell cycle arrest of the MSCs. Meanwhile, compared to the mono-cultured MSCs, the gene expression profiles of MSCs in the leukemia niche changed significantly. These differential genes were enriched for cell cycle, cell differentiation, DNA replication, as well as some tumor-promoting biofunctions including protein phosphorylation, cell migration and angiogenesis. Further, interferon alpha-inducible protein 6 (IFI6), as a gene activated by interferon, was highly expressed in leukemia niche MSCs. The leukemia cell multiplication was facilitated evidently by IFI6 both in vitro and in vivo. Mechanistically, IFI6 might promote leukemia cell proliferation by stimulating SDF-1/CXCR4 axis, which leads to the initiation of downstream ERK signaling pathway. As suggested by further RNA sequencing analysis, the high IFI6 level in MSCs somewhat influenced the gene expression profile and biological functions of leukemia cells.

**Conclusions:**

BM-MSCs in the leukemia niche have varying degrees of changes in biological characteristics and gene expression profiles. Overexpression of IFI6 in BM-MSCs could be a key factor in promoting the proliferation of B-ALL cells, and this effect might be exerted through the SDF-1/CXCR4/ERK signal stimulation. Targeting IFI6 or related signaling pathways might be an important measure to reduce the leukemia cell proliferation.

**Supplementary Information:**

The online version contains supplementary material available at 10.1186/s12967-023-04464-1.

## Background

As a malignant hematological condition, adult B-cell acute lymphoblastic leukemia (B-ALL) is caused by the abnormal proliferation of B-lymphocyte lineage precursor cells in the bone marrow. Studies have shown that complete remission is achievable in most patients with B-ALL after receiving induction chemotherapy [1, 2]. However, the recurrence, drug resistance and extramedullary infiltration during treatment remain the chief reasons for the poor efficacy in B-ALL patients, and the long-term efficacy is not optimistic [3, 4].

The tumor microenvironment (TME) is a dynamic network, which can offer a supportive environment for the emergence and development of tumor cells [5]. Lately, the indispensable and decisive role of TME in tumor progression has attracted a growing scholarly attention [6, 7]. During the tumorigenesis and evolution, tumor cells exist in close proximity to their surrounding stromal microenvironment, and due to the extensive “cross-talks” between them, TME can promote their survival and metastasis [8, 9].

As a kind of crucial stromal cells in the TME, bone marrow mesenchymal stem cells (BM-MSCs) are often important factors in protecting leukemia cells from chemotherapeutics and promoting their migration and invasion [10–12]. However, in vitro experiments, previous reports have shown the presence of certain differences between MSCs cultured alone and MSCs in a leukemia niche [13, 14], and the later could be simulated by constructing a co-culture system of leukemia cells and BM-MSCs in vitro [13]. To our knowledge, the differences in biological functions and gene expression profiles between mono-cultured MSCs and MSCs in B-ALL-derived leukemia niches remain unclear in some respects.

In the present work, it was revealed that MSCs in the leukemia niche have varying degrees of changes in biological characteristics and gene expression profiles. Through differentially expressed genes (DEGs) analysis, we focused on the abnormal expression of interferon alpha-inducible protein 6 (IFI6), which is a gene activated by interferon capable of facilitating the tumorigenesis and development of several solid tumors [15–17]. We elucidated IFI6's positive role in the leukemia cell multiplication, and explored the underlying molecular mechanisms. These findings perhaps offer a novel theoretical and experimental foundation for unraveling the leukemia evolution-facilitating mechanism of the microenvironment, and for finding potential therapeutic targets.

## Methods

### Cell cultures

Human ALL (Nalm-6 and RS4;11) cells, which were acquired from the Laboratory of Hematopoietic Stem Cell Transplantation Center of Guizhou Province (Guiyang, China), were cultured in a RPMI-1640 medium involving FBS (10%), streptomycin (100 mg/mL) and penicillin (100 units/mL) at 37 °C with 5% CO_2_.

For the acquisition of BM-MSCs, we separated BM-MSCs from the bone marrow aspirates of B-ALL patients (n = 37) with their consent. The aspirates were subjected to ficoll gradient centrifugation, and subsequently cultured in the complete medium of adult BM-MSCs (Saiye, Shanghai, China). The phenotypic traits and differentiation capacities of BM-MSCs have been confirmed by us priorly [18].

### Co-culture system and establishment of leukemia niche in vitro

For the construction of co-culture system, the culture plate was initially seeded with BM-MSCs at 1 × 10^5^/ml for 24 h, and then added with leukemia cells at a 4:1 ratio. For the establishment of the leukemia niche, MSCs were subjected to a 72-h co-cultivation with leukemia cells with reference to previously published literature [13]. In each independent repeated experiment, separately cultured MSCs from the same patient as control. For the collection of suspended leukemia cells in the co-culture system, Nalm-6/RS4;11 cells were pipetted cautiously from the monolayer MSCs. For some leukemia cells adhering to MSCs surface, PBS-EDTA 1 was used to wash the co-culture extensively to remove all the leukemia cells.

### Reagents and antibodies

Vincristine sulfate, which was product of Taoshu Biotechnology (Shanghai, China), was prepared in phosphate buffer solution (PBS). AMD3100 and PD98059 from MedChemExpress (Shanghai, China) were formulated separately in dimethyl sulfoxide (DMSO) and anhydrous ethanol. The AKT, ERK, phospho-AKT and phospho-ERK antibodies were procured from Cell Signaling Technology (Danvers, MA, USA)., while the CXCR4 and IFI6 antibodies were procured separately from Solibao Biotechnology (Beijing, China) and ImmunoWay Biotechnology (Plano, TX, USA). Proteintech Group (Wuhan, China) was the provider of the secondary antibody used herein for Western blot.

### Cell proliferation

MSCs (500 ul) were seeded in a 24-well culture plate at 5 × 10^4^/ml. After the MSCs adhered overnight, the MSCs culture medium was aspirated, and 500 ul of Nalm-6/RS4;11 cells (1-3 × 10^5^/ml) were inoculated on MSCs. The mono-cultured Nalm-6/RS4;11 cells were set as a control, where RPMI 1640 medium was used as a culture medium. The leukemia cells in each well were collected after incubation for 24, 48, 72 and 96 h, respectively. Then, the leukemia cells were quantified by direct counting using a cell counter, and the cell growth curves were drawn according to the results.

### Apoptosis and cell cycles

After harvesting and PBS-washing, the cells were subjected to Annexin-V/propidium iodide (PI) staining to assay the apoptotic ratio as per the advised protocol (7Sea Pharmatech, Shanghai, China). To assess the cell cycle, RNase A and PI (7Sea Pharmatech, Shanghai, China) were utilized to treat the gathered MSCs, followed by flow cytometry (BD Biosciences, San Jose, USA).

### Cell migration and invasion

Matrigel-coated and uncoated Transwell chambers were used for invasion and migration experiments, respectively. The lower chamber was added with 650 ul of MSCs (1 × 10^5^/mL) and incubated overnight. Next, the upper chamber (pore size: 8.0 um, Corning Incorporated, Costar) was added with 100 ul of Nalm-6/RS4; 11 cells (4 × 10^5^/mL) and subjected to a 24-h incubation. An inverted microscope was utilized to surveil the leukemia cell migration and invasion in the lower chamber, followed by photographing under 40 × magnification. Quantification of migrated and invaded cells was accomplished by direct counting using a cell counter.

### β-galactosidase staining

For adherent BM-MSCs, after washing with PBS in a 6-well plate, the procedure for β-galactosidase staining was implemented as per the specific guidelines. then followed by observation of the proportion of cells stained blue (senescent cells) under a microscope and photographing (200 ×). The proportion of senescent cells in each group was evaluated with 5 random fields of view, and then the senescence of cells in each group was assessed by value averaging.

### Lentiviral transduction

Human IFI6-silencing RNA (si-IFI6) and IFI6-overexpressing clone lentiviral particles (LV-IFI6) were the Genechem (Shanghai, China) products. Transfection of si-IFI6/LV-IFI6 was accomplished as per the manufacturer protocol. Controls used were the empty vector (EV)-transfected BM-MSCs.

### Quantitative real-time PCR

Trizol reagent (Qiagen, Hilden, Germany) was utilized to extract the total RNA of cells, the FastKinggDNA Dispelling RT SuperMix (Qiagen, Hilden, Germany) was utilized to reversely transcribe the RNA extract to cDNA. Then, cDNA was analyzed by quantitative real-time PCR (qRT-PCR) in accordance with the protocols of primers and Talent qPCR PreMix (SYBR Green) (Qiagen, Hilden, Germany). For the target gene, their relative expression levels were estimated by the comparative CT (2^−△CT^) approach following normalization to β-actin. Table 1 details the human primers (Generay Bioteach, Shanghai, China) used.

**Table 1:** Primer sequences used for real-time PCR

Primer name	Sequence(5'-3')
h*MX-1* (F)	GTTTCCGAAGTGGACATCGCA
h*MX-1* (R)	CTGCACAGGTTGTTCTCAGC
h*IFITM1* (F)	CCAAGGTCCACCGTGATTAAC
h*IFITM1* (R)	ACCAGTTCAAGAAGAGGGTGTT
h*IFIT3* (F)	AAAAGCCCAACAACCCAGAAT
h*IFIT3* (R)	CGTATTGGTTATCAGGACTCAGC
h*ISG15* (F)	CGCAGATCACCCAGAAGATCG
h*ISG15* (R)	TTCGTCGCATTTGTCCACCA
h*IFI6* (F)	GGTCTGCGATCCTGAATGGG
h*IFI6* (R)	TCACTATCGAGATACTTGTGGGT
hIFI44L (F)	ACAGAGCCAAATGATTCCCTATG
hIFI44L (R)	TCGATAAACGACACACCAGTTG
h*IFIT1* (F)	AGAAGCAGGCAATCACAGAAAA
h*IFIT1* (R)	CTGAAACCGACCATAGTGGAAAT
h*P-53* (F)	CTGCCCTCAACAAGATGTTTTG
h*P-53* (R)	CTATCTGAGCAGCGCTCATGG
h*P-21* (F)	GCCTGGACTGTTTTCTCTCG
h*P-21* (R)	ATTCAGCATTGTGGGAGGAG
h*P-16*(F)	GAAGGTCCCTCAGACATCCCC
h*P-16* (R)	CCCTGTAGGACCTTCGGTGAC
h*SOX2* (F)	GCCGAGTGGAAACTTTTGTCG
h*SOX2* (R)	GGCAGCGTGTACTTATCCTTCT
h*NANOG* (F)	TTTGTGGGCCTGAAGAAAACT
h*NANOG* (R)	AGGGCTGTCCTGAATAAGCAG
h*ADIPOQ* (F)	AACATGCCCATTCGCTTTACC
h*ADIPOQ* (R)	TAGGCAAAGTAGTACAGCCCA
h*PPAR-γ* (F)	GGGATCAGCTCCGTGGATCT
h*PPAR-γ* (R)	TGCACTTTGGTACTCTTGAAGTT
h*RUNX2* (F)	CCGCCTCAGTGATTTAGGGC
h*RUNX2* (R)	GGGTCTGTAATCTGACTCTGTCC
h*BGLAP* (F)	CACTCCTCGCCCTATTGGC
h*BGLAP* (R)	CCCTCCTGCTTGGACACAAAG
h*RUNX1* (F)	CTGCCCATCGCTTTCAAGGT
h*RUNX1* (R)	GCCGAGTAGTTTTCATCATTGCC
h*HOXB4* (F)	CGTGAGCACGGTAAACCCC
h*HOXB4* (R)	CGAGCGGATCTTGGTGTTG
h*POU5F1* (F)	CTTGAATCCCGAATGGAAAGGG
h*POU5F1* (R)	GTGTATATCCCAGGGTGATCCTC
h*SDF-1* (F)	CACTTTAGCTTCGGGTCAATG
h*SDF-1* (R)	ACACTCCAAACTGTGCCCTTCA
h*β-actin* (F)	CTACCTCATGAAGATCCTCACCGA
h*β-actin* (R)	TTCTCCTTAATGTCACGCACGATT

*F* forward, *R* reverse

### Western blotting

Cells were collected and lysed with Radio ImmunoprecipitationAssay (RIPA) lysis buffer (Beyotime, Shanghai, China) involving phenylmethylsulfonyl fluoride (PMSF; 1%). Protein (10–30 μg) was isolated on SDS-PAGE and then shifted onto the PVDF membrane. A 1–2 h blockade of the membrane proceeded using skimmed milk (5%) at room temperature, followed by an overnight incubation using primary antibodies at 4 ℃. Thereafter, an extra 1-h incubation of the membranes was accomplished using secondary antibody at ambient temperature, and then the protein expression was assayed with electrochemiluminescence reagent. Gray value analysis was performed via the Image J software against the internal β-actin reference.

### Transcriptome sequencing analysis

For transcriptome sequencing analysis, the processed MSCs and leukemia cells were collected respectively. After cells were lysed by trizol reagent, the samples were sent to Shanghai Liebing Information Technology and Hangzhou Lianchuan Biotechnology for transcriptome sequencing analyses. Meanwhile, the gene expression profile dataset GSE101454 related to MSCs derived from patients with B-Cell Precursor ALL (BCP-ALL) in GEO database was selected. This dataset provided microarray analysis data of mono-cultured BM-MSCs and BCP-ALL cells- co-cultured BM-MSCs (for 40 h).

### Analysis of gene datasets and screening of DEGs

The DEGs in the datasets were analyzed and screened, where the screening conditions were: |log2FC|> 1, Flase Discovery Rate (FDR) < 0.05, P < 0.05. During the experimentation, Gene Ontology (GO) analysis and Kyoto Encyclopedia of Genes and Genomes (KEGG) pathway enrichment were performed on the DEGs via the Database For Annotation Visualization and Integrated Discovery (DAVID) software, and the biological functions and signaling pathway changes enriched by the differential genes were screened out. Gene set enrichment analysis (GSEA) 4.2.3 was utilized to assess the enrichment of gene sets.

### Xenografted tumor model

Xenograft assays in mice were approved by the Guizhou Medical University's Animal Care Welfare Committee. 4–6 weeks-old female nonobese diabetes/severe combined immunodeficiency (NOD/SCID) mice were chosen, each of which was given subcutaneous injection of RS4;11/Nalm-6 cells, a RS4;11/Nalm-6-MSC mixture, a RS4;11/Nalm-6-MSCs-EV mixture, and a RS4;11/Nalm-6-MSCs-LV-IFI6 mixture (1 × 10^6^ MSCs mixed with 4 × 10^6^ RS4;11/Nalm-6 cells) into the left chest. Growth of tumors was surveilled every 3 d through length (L) and width (W) determination, and the computational formula for tumor volume was: 0.5 LW^2^. After injection for 25d/34d, the tumor tissues were extracted from mice and embedded in paraffin for further study. The procedure for immunohistochemistry (IHC) staining was implemented as per the specific guidelines. The primary and secondary antibodies used were 1:100 dilutions.

### Statistical analysis

SPSS 20.0 was utilized to assess the data. Independent student's t-test was employed for making two groups comparison, while one-way ANOVA was adopted for homogeneity of variance assessment among multiple groups. Non-normal data were subjected to the Kruskal–Wallis non-parametric test. P-values were indicated as follows: *P < 0.05;**P < 0.01; ***P < 0.001.

## Results

### MSCs in leukemia niche exhibited diverse alterations in biological characteristics

**Fig. 1:**
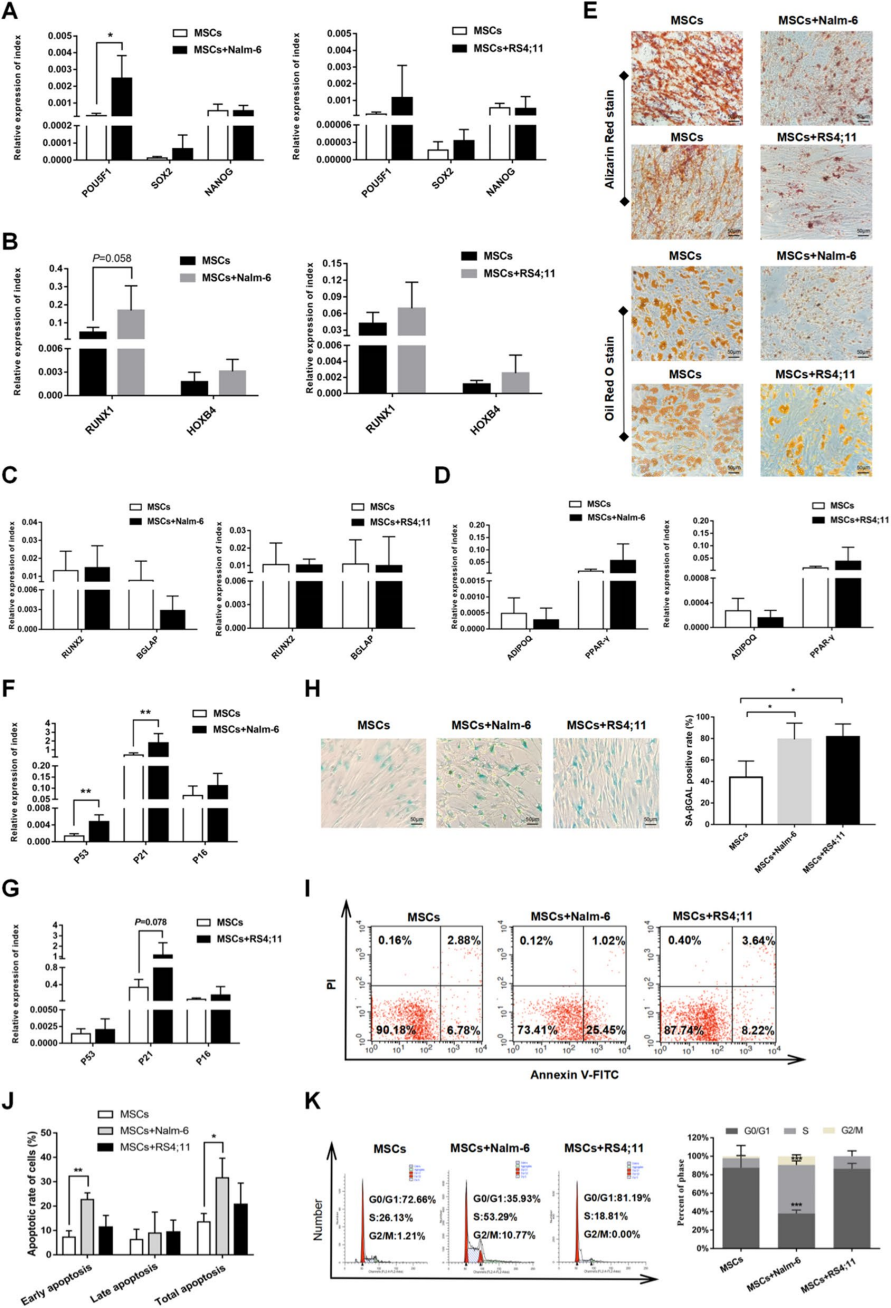
MSCs in the leukemia niche exhibited diverse alterations. **A** The expression levels of POU5F1, SOX2 and NANOG in MSCs cultured alone group and MSCs co-cultured with Nalm-6/RS4;11 group for 72 h by qRT-PCR, n = 6 independent experiments. **B** The expression levels of RUNX1 and HOXB4 in MSCs cultured alone group and co-cultured with Nalm-6/RS4;11 group, n = 6.** C** and** D** The expression levels of the associated indicators of osteogenic (RUNX2, BGLAP) and adipogenic (ADIPOQ, PPAR-γ) differentiation in MSCs cultured alone group and co-cultured with Nalm-6/RS4;11 group, n = 6.** E** Representative images of Alizarin Red stained MSCs in cultured alone and co-culture with Nalm-6/RS4;11 group following 21 days of osteogenic induction and Oil Red O stain following 15 days of adipogenic induction (200 × , scale bars, 50 µm). **F** and **G** The expression levels of p53, p21 and p16 in MSCs cultured alone and co-cultured with Nalm-6/RS4;11, n = 6. **H** Senescence-associated β-galactosidase staining in MSCs cultured alone and co-cultured with Nalm-6/RS4;11 (200 × , scale bars, 50 µm), n = 3.** I** and** J** Percentages of apoptotic MSCs cultured alone and co-cultured with Nalm-6/RS4;11 for 72 h, n = 3. **K** Cell cycle arrest in MSCs cultured alone and co-cultured with Nalm-6/RS4;11 for 72 h, n = 3. Each value indicates the mean ± standard deviation (SD) of three or more independent experiments. *P < 0.05, **P < 0.01, ***P < 0.001

To explore the possible differences between MSCs in the leukemia niche (MSCs co-culture with leukemia cells for 72 h) and MSCs cultured alone, we compared these two MSC groups regarding cell stemness, self-renewal, senescence, adipogenic and osteogenic differentiation, cell cycle, as well as cell apoptosis. In terms of cell stemness-associated indicators (POU5F1, SOX2, NANOG), POU5F1 was found to be significantly increased in the MSC–Nalm-6 co-culture system, but no obvious changes were seen in the expressions of SOX2 and NANOG (Fig. 1A). For the self-renewal-associated indicators (RUNX1, HOXB4), the markers of adipogenic (ADIPOQ, PPAR-γ) and osteogenic differentiation (RUNX2, BGLAP), the changes between the two groups were not significant (Fig. 1B, C and D). However, when MSCs co-cultured for 72 h with Nalm-6/RS4;11 were subjected to adipogenic and osteogenic differentiation experiments, the differentiation abilities of co-cultured MSCs were weakened compared to the mono-cultured MSCs (Fig. 1E).

Regarding the cell senescence-related markers, compared to the mono-cultured MSCs, the expressions of p53, p21 and p16 in the co-cultured MSCs presented upward trends, and the change in p21 was more obvious (Fig. 1F, G). On the basis of the foregoing results, the changes of cell senescence between the two groups were further observed by senescence-associated β-galactosidase staining, which found markedly increased senescence degree of co-cultured MSCs compared to that of mono-cultured ones (Fig. 1H). Suggestively, the MSCs in the leukemia niche might have varying degrees of weakened differentiation abilities and senescence changes.

Next, we further explored the possible changes in the apoptosis and cell cycle of co-cultured MSCs. Through testing, we found that in the MSC–Nalm-6 co-culture system, MSCs underwent a certain degree of apoptosis (Fig. 1I, J), and exhibited pronouncedly more S phase cells and declined proportion of G0/G1 phase cells, but no similar changes were observed in MSCs in the RS4;11 co-culture system (Fig. 1K). Based on the above results, we speculated that the biological characteristics of MSCs in the leukemia niche might have changed to varying degrees.

### MSCs in the leukemia niche exhibited altered gene expression profiles

**Fig. 2:**
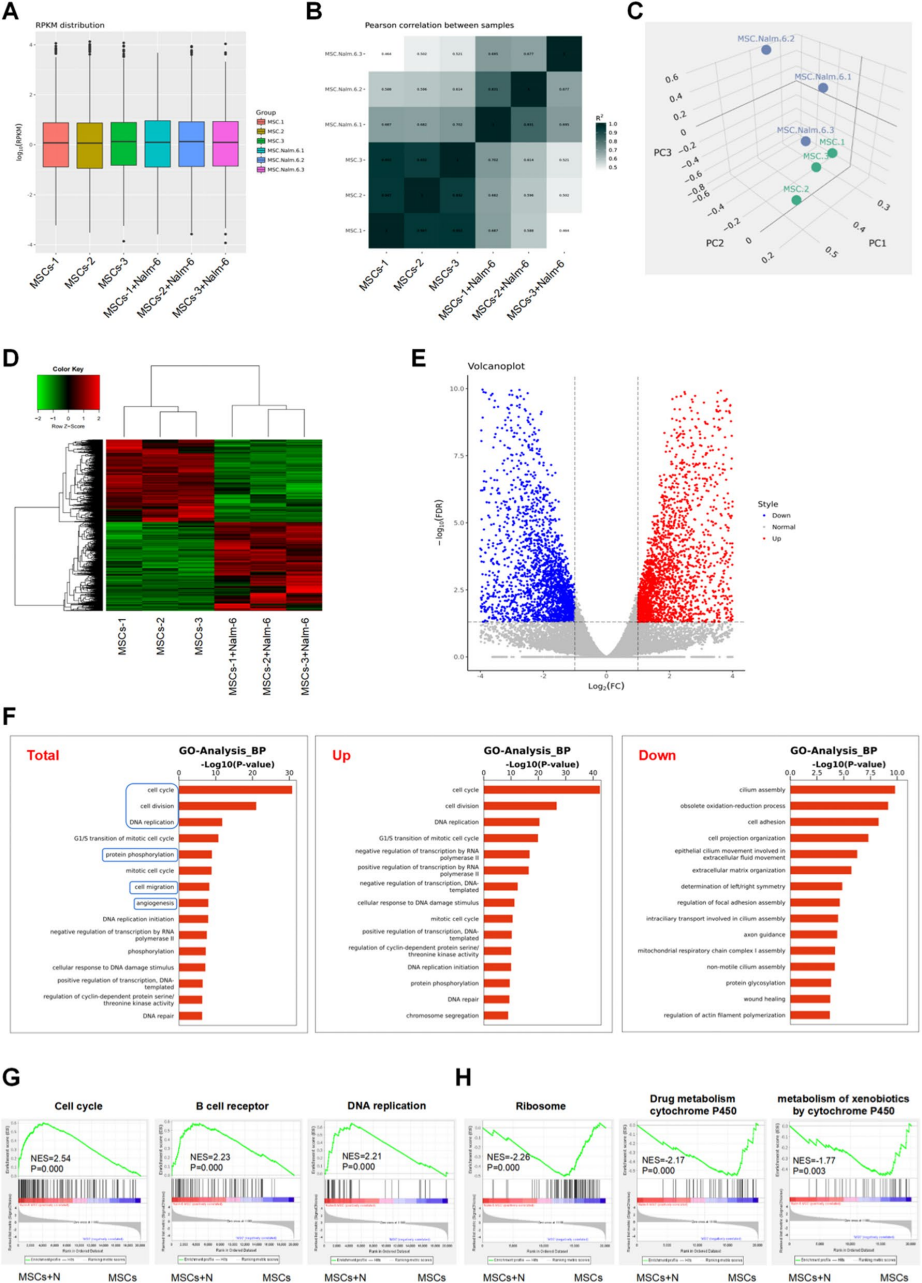
MSCs in the leukemia niche showed altered gene expression profiles. **A** The distributions of gene expression in MSCs cultured alone and MSCs co-cultured with Nalm-6 for 72 h based on log_10_(RPKM), (n = 3 independent experiments). **B** Correlation analysis of MSCs cultured alone and MSCs co-cultured with Nalm-6 by HeatMap diagram. **C** Principal component analysis of MSCs cultured alone and MSCs co-cultured with Nalm-6. **D** and **E** The heatmap (**D**) and volcano plot (**E**) of gene expression in MSCs cultured alone and co-cultured with Nalm-6 based on -log_10_(FDR) (MSCs + Nalm-6 vs MSCs). **F** GO-Biological Process analysis of total differentially expressed genes (Total), upregulated genes (UP) and downregulated genes (Down) in MSCs co-cultured with Nalm-6 group compared to MSCs cultured alone group. **G** and** H** GSEA of differentially functional gene subsets in MSCs co-cultured with Nalm-6 (**G**) and MSCs cultured alone (**H**)

In the following experiments, we further compared the molecular biological changes between the two groups by transcriptome sequencing analysis. Given the more obvious changes of Nalm-6-co-cultured MSCs in the previous detection, we selected the MSCs co-cultured with Nalm-6 for 72 h for sequencing analysis. Figure 2A, B and C separately illustrate the distributions of gene expressions in the tested samples (Fig. 2A), the correlations between samples (Fig. 2B), as well as the clustering features of gene expression patterns (Fig. 2C). Through differential gene analysis, it was found that 5,543 of 20,097 gene expression profiles detected were expressed differentially in MSCs in the co-culture system (|log_2_FC|> 1, FDR < 0.05), with 2,874 up-regulated genes and 2,669 down-regulated genes (Fig. 2D, E). As revealed by further GO analysis, the DEGs in the co-cultured MSCs were implicated in biofunctions like cell division, cell cycle and DNA replication, and were also involved in several tumor-promoting biofunctions such as protein phosphorylation, cell migration and angiogenesis (Fig. 2F). Through a concurrent GSEA, we discovered the collective enrichment of particular datasets, such as positive cell cycle regulation, B cell receptor signaling pathway and DNA replication, in the co-culture group (Fig. 2G). Contrastively, representative enrichment of subsets like ribosome, drug metabolism cytochrome P450 and metabolism of xenobiotics by cytochrome P450 was noted in MSCs mono-culture group (Fig. 2H). These results suggested that MSCs in leukemia niches have different gene expression profiles and biological functions, including several functions that promote tumor progression.

### IFI6 was abnormally expressed in MSCs in the leukemia niche through screening the gene expression profile

**Fig. 3:**
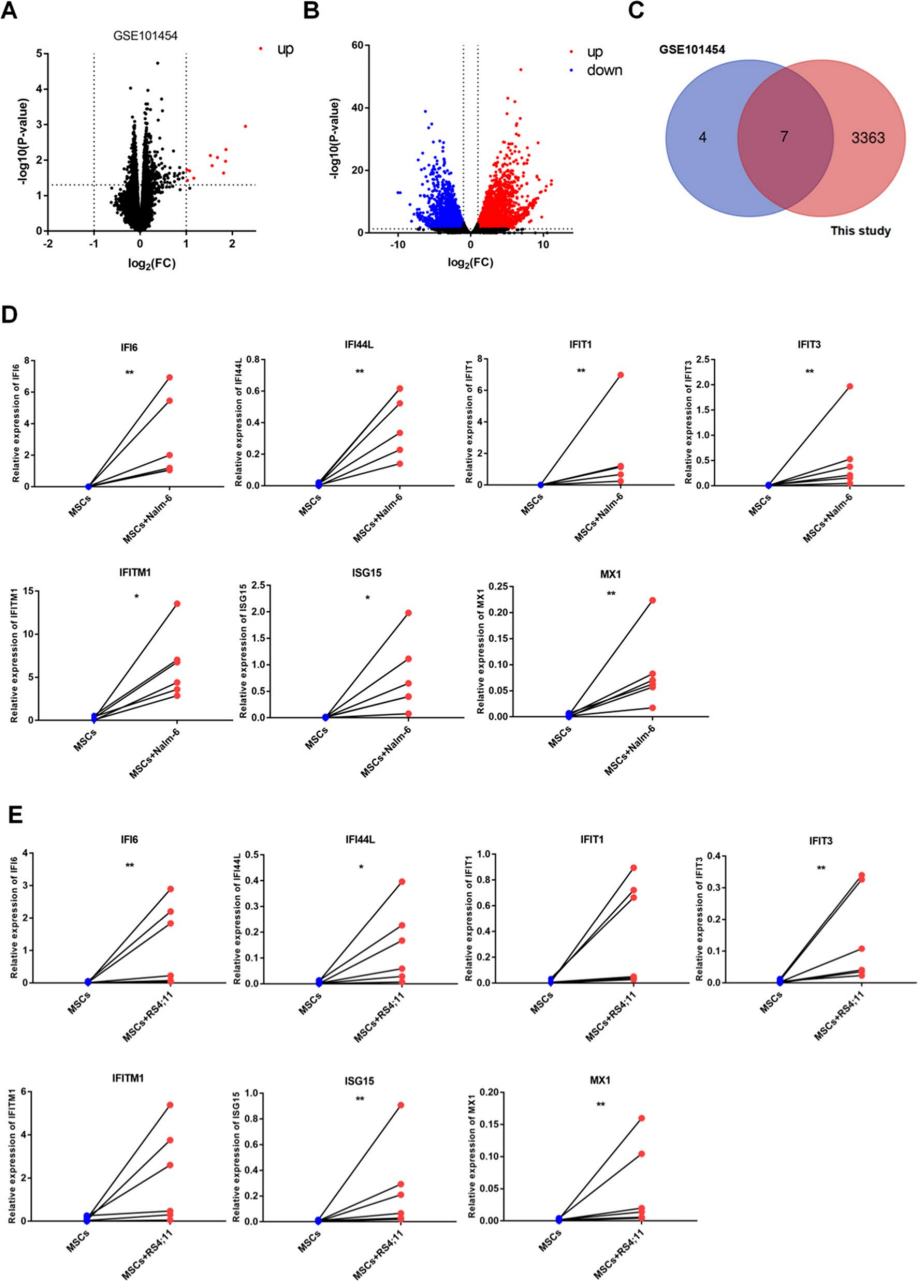
The gene expression profiling datas were been screened. **A** and **B** The volcano plots of gene expression in GSE101454 dataset (**A**) and this study (**B**) based on -log_10_ (P value) (MSCs in co-culture vs MSCs in mono-culture). **C** Venn diagram for differentially expressed genes visualization in GSE101454 dataset and this study. **D** and** E** The expression levels of IFI6, IFI44L, IFIT1, IFIT3, IFITM1, ISG15 and MX1 in MSCs cultured alone group and MSCs co-cultured with Nalm-6 (**D**) or RS4;11 (**E**) group for 72 h by qRT-PCR. Each value indicated three or more independent experiments. *P < 0.05, **P < 0.01

In the experiments below, further emphasis was placed on the DEGs in MSCs in the leukemia niche. We searched the Gene Expression Omnibus (GEO) database and screened a dataset (GSE101454) with a certain degree of similarity to our study. Through DEG analysis on this dataset, 11 DEGs were screened out (|log_2_FC|> 1, P < 0.05), all of which were up-regulated genes in co-culture system (Fig. 3A). Subsequently, the data from gene expression profiling in the present work were assessed by the same condition setting (Fig. 3B). We performed an intersection analysis on the DEGs in the two gene expression profiling datas and drew a Venn diagram (http://bioinformatics.psb.ugent.be/webtools/Venn/) (Fig. 3C), founding that a total of 7 genes generated intersections, namely MX1, IFITM1, IFIT3, ISG15, IFI6, IFI44L and IFIT1. Based on these results, the expression of these 7 genes in the mono-cultured and co-cultured MSCs were further examined via qRT-PCR, finding varying degrees of elevations in the 7 genes in the co-culture system (Figs. 3D, E). By comparing the up-regulated folds, it was found that the IFI6 level in the co-cultured MSCs had the highest fold increase (Table 2).

**Table 2:** The fold change of the expression of the seven differentially expressed genes in MSCs in the co-culture system

Index	Fold change relative to MSCs culture alone group (Mean ± standard error)	
	MSCs + Nalm-6	MSCs + RS4;11
MX1	52.7995 ± 26.78334	19.4819 ± 7.87327
IFITM1	57.7066 ± 28.48986	28.7781 ± 14.27821
IFIT3	42.6124 ± 16.26199	31.6125 ± 7.18548
ISG15	85.9352 ± 43.63573	31.6485 ± 11.79391
IFI6	197.1879 ± 62.68515	154.3834 ± 120.35351
IFI44L	60.6177 ± 26.00720	38.0550 ± 18.26454
IFIT1	102.9635 ± 30.00929	41.6613 ± 20.31445

### Increased IFI6 expression in leukemia niche promoted the proliferation of B-ALL cells

**Fig. 4:**
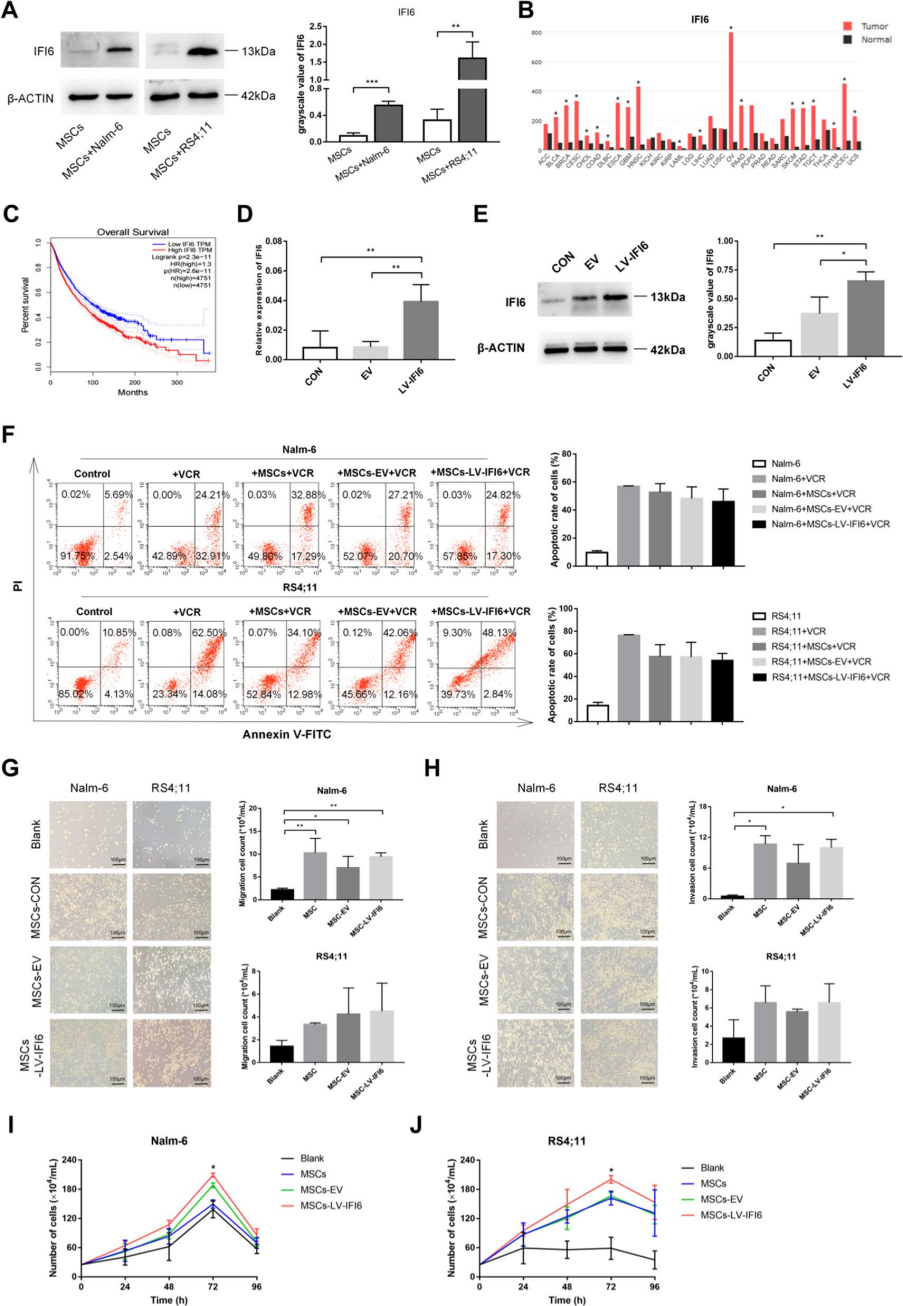
The increased expression of IFI6 in MSCs in leukemia niche promotes the proliferation of B-ALL cells. **A** Western blot was used to detect the expression levels of IFI6 in MSCs cultured alone and co-cultured with Nalm-6/RS4;11 for 72 h, mean ± SD. **B** The expression of IFI6 in normal tissues and tumor tissues through the analysis of GEPIA database. **C** The survival analysis of IFI6 in normal tissues and tumor tissues through GEPIA database. **D** and **E** The mRNA and protein levels of IFI6 in MSCs with control group (CON), empty group (EV) and up-regulated IFI6 group (LV-IFI6), mean ± SD. **F** Percentages of apoptotic Nalm-6 and RS4;11 cells co-cultured with blank, MSCs, MSCs-EV and MSCs-LV-IFI6 after vincristine sulfate treatments (2.5 μM for Nalm-6; 5 μM for RS4; 11) for 24 h, mean ± SD. **G** and **H** The numbers of migratory (**G**) and invasive (**H**) Nalm-6/RS4;11 cells co-cultured with blank, MSCs-CON, MSCs-EV and MSCs-LV-IFI6 groups after 24 h of cells incubation (40 × , scale bars, 100 µm), mean ± SD. **I** and** J** The numbers of cell proliferation of Nalm-6 and RS4;11 cells co-cultured with blank, MSCs, MSCs-EV and MSCs-LV-IFI6 groups after 24 h, 48 h, 72 h and 96 h of cells incubation, mean ± SEM, the “*” in the figure represents a significant difference between the MSCs-LV-IFI6 and MSCs-EV groups. Each value indicated three or more independent experiments. *P < 0.05, **P < 0.01, ***P < 0.001

According to the above results, we further verified the difference in the IFI6 expression in mono-cultured and co-cultured MSCs via western blot. Prominently higher IFI6 level was noted in the co-cultured MSCs (Fig. 4A). To explore whether the increased expression of IFI6 has some biological effect, we conducted preliminary exploration through the GEPIA database (http://gepia.cancer-pku.cn/detail.php) to assess the normal and tumor tissue levels of IFI6, discovering obviously increased IFI6 levels in several tumors (Fig. 4B). As revealed by survival analysis, high IFI6 level was linked to lower rate of overall survival (Fig. 4C). Thus, we further explored the potential impact of IFI6 in MSCs on the biological function of B-ALL cells. Initially, the expression of IFI6 in MSCs was up-regulated by lentiviral transfection reagent. The fluorescence microscopy (Additional file 1: Figure S1A), qRT-PCR and western blot was employed to validate the transfection efficiency (Fig. 4D, E). By functional testing, results showed that the high expression of IFI6 in MSCs had a slight effect on the changes of sensitivity of leukemic cells to vincristine (Fig. 4F), migration and invasiveness of leukemia cells (Fig. 4G, H), but exerted a pro-proliferative effect on the leukemia cells (Fig. 4I, J). Further down-regulation of IFI6 in MSCs (Additional file 1: Figure S1B, Additional file 2: Figure S2A, B) led to the opposite results in cell proliferation (Additional file 2: Figure S2C, D). As implied by these findings, the elevated IFI6 in MSCs might promote the proliferation of leukemia cells.

### Overexpression of IFI6 in MSCs promoted the growth and proliferation of leukemia cells in vivo

**Fig. 5:**
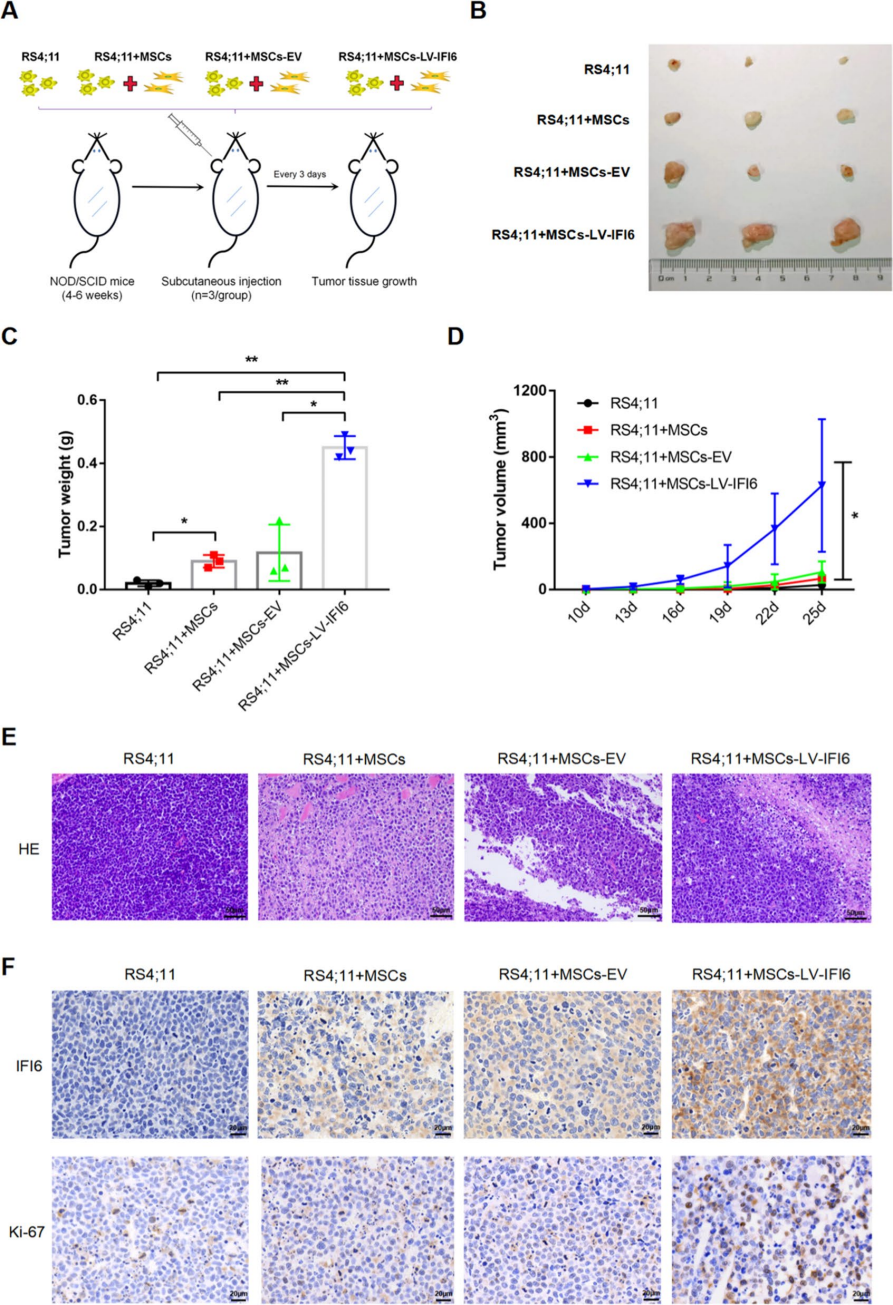
IFI6 promotes the growth and proliferation of RS4;11 cells in vivo. **A** Schematic diagram of subcutaneous tumor formation in mice. **B** The size of subcutaneous tumors in the RS4;11 injection group, RS4;11 + MSCs injection group, RS4;11 + MSCs-EV injection group, and RS4;11 + MSCs-LV-IFI6 injection group, n = 3. **C** Average tumor weight in each group was calculated at 25 days after injection. mean ± SD. *P < 0.05, **P < 0.01. **D** Average tumor volume in each group was evaluated at 25 days after injection. mean ± SD. *P < 0.05. **E** HE staining (200 ×) of tumor tissue in each group (scale bars, 50 µm). **F** The expression of IFI6 (400 ×) and Ki-67 (400 ×) in xenotransplanted tumors in each group with immunohistochemical staining (scale bars, 20 µm)

In the following experiments, we further applied mice with NOD/SCID to investigate IFI6's role in the growth and proliferation of leukemia cells. We subcutaneously injected RS4;11 (n = 3) and a suspension of RS4;11 and MSCs/MSCs-EV/MSCs-LV-IFI6 (4:1) (n = 3) into the left chest of mice, respectively, and then observed the growth of tumor tissues (Fig. 5A). According to the results, the volume and mass of tumor tissues in the RS4;11 + MSCs-LV-IFI6 group were obviously increased in contrast to the RS4;11, RS4;11 + MSCs and RS4;11 + MSCs-EV groups (Fig. 5B, C and D). In addition, in vivo experimental validation of Nalm-6 cell lines yielded similar results (Additional file 3: Figure S3A, B, C and D). Figure 5E, F displayed the hematoxylin–eosin (HE) staining results and the expressions of IFI6 and Ki-67 in various groups, more IFI6 and Ki-67 expression was observed in tumor tissues of the RS4;11 + MSCs-LV-IFI6 group compared with the RS4;11, RS4;11 + MSCs and RS4;11 + MSCs-EV groups. These datas suggested that high expression of IFI6 in MSCs might promote the leukemia cell growth and multiplication in vivo.

### IFI6 promoted the proliferation of B-ALL cells by stimulating the SDF-1/CXCR4 axis to activate the ERK signaling pathway

**Fig. 6:**
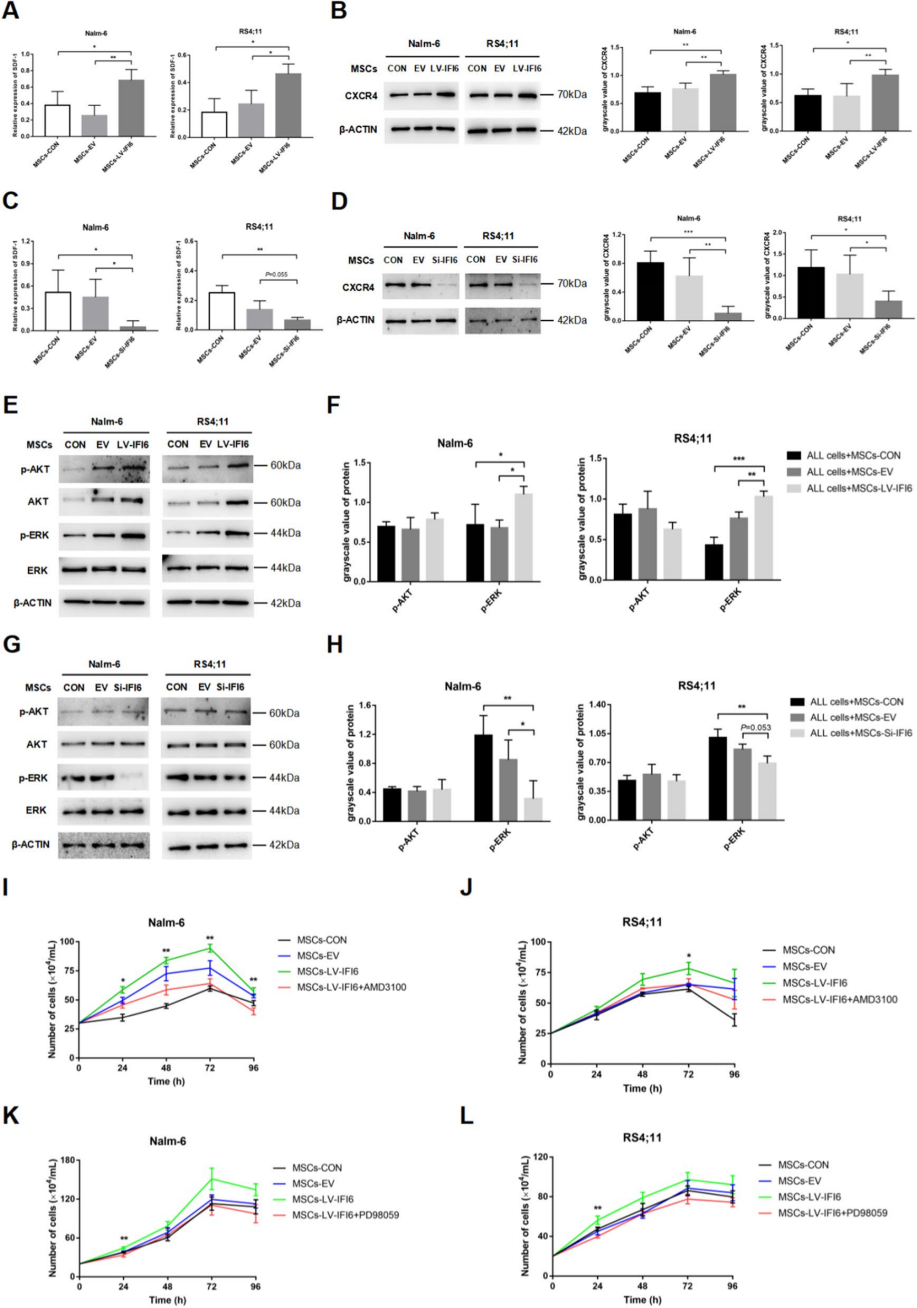
IFI6 promotes the proliferation of B-ALL cells by stimulating the SDF-1/CXCR4 axis to activate the ERK signaling pathway. **A** The expression levels of SDF-1 in MSCs with CON, EV and LV-IFI6 co-cultured with Nalm-6 (n = 4) /RS4;11 (n = 3) for 72 h, mean ± SD. **B** The expression levels of CXCR4 in Nalm-6/RS4;11 co-cultured with MSCs-CON, MSCs-EV and MSCs-LV-IFI6 for 72 h, mean ± SD, n = 4. **C** The expression levels of SDF-1 in MSCs with CON, EV and Si-IFI6 co-cultured with Nalm-6/RS4;11 for 72 h, mean ± SD, n = 4. **D** The expression levels of CXCR4 in Nalm-6/RS4;11 co-cultured with MSCs-CON, MSCs-EV and MSCs-Si-IFI6 for 72 h, mean ± SD, n = 4. **E** and** F** The expression levels of p-AKT and p-ERK in Nalm-6/RS4;11 co-cultured with MSCs-CON, MSCs-EV and MSCs-LV-IFI6 for 72 h, mean ± SD, n = 3. **G** and** H** The expression levels of p-AKT and p-ERK in Nalm-6/RS4;11 co-cultured with MSCs-CON, MSCs-EV and MSCs-Si-IFI6 for 72 h, mean ± SD, n = 3. **I** and **J** The numbers of cell proliferation of Nalm-6 and RS4;11 cells co-cultured with MSCs-CON, MSCs-EV, MSCs-LV-IFI6 and MSCs-LV-IFI6 + AMD3100 (20 μM) groups after 24 h, 48 h, 72 h and 96 h of cells incubation, mean ± SEM, n = 3, the “*” in the figure represents a significant difference between the MSCs-LV-IFI6 and MSCs-LV-IFI6 + AMD3100 groups. **K** and **L** The numbers of cell proliferation of Nalm-6 and RS4;11 cells co-cultured with MSCs-CON, MSCs-EV, MSCs-LV-IFI6 and MSCs-LV-IFI6 + PD98059 (20 μM) groups after 24 h, 48 h, 72 h and 96 h of cells incubation, mean ± SEM, n = 4, the “*” in the figure represents a significant difference between the MSCs-LV-IFI6 and MSCs-LV-IFI6 + PD98059 groups. *P < 0.05, **P < 0.01, ***P < 0.001

Next, we aimed to explore the underlying mechanism by which IFI6 promotes leukemia cell proliferation. Stromal cell derived factor 1 (SDF-1)/chemokine receptor 4 (CXCR4) is an important signaling axis that mediates the communication between tumor and stromal cells. We speculated whether the increased expression of IFI6 in MSCs could facilitate the leukemia cell multiplication by stimulating the SDF-1/CXCR4 axis. Through the expression comparison for SDF-1 in co-cultured MSCs between the EV, LV-IFI6 and control groups, a prominent elevation of SDF-1 level was noted in the MSCs of the LV-IFI6 group (Fig. 6A). Based on this result, the level of CXCR4, the SDF-1 receptor, in leukemia cells was further examined, finding that in the co-culture system, up-regulation of IFI6 could significantly increase the leukemia cell level of CXCR4 (Fig. 6B), while down-regulation of IFI6 yielded the opposite results (Fig. 6C, D). In subsequent experiments, we further detected the proliferative signaling pathways of AKT and ERK downstream of the SDF-1/CXCR4 axis in leukemia cells. Evidently elevated p-ERK level was noted in the IFI6 up-regulated group, while the levels of p-AKT and AKT both presented upward trends (Fig. 6E, F). However, down-regulation of IFI6 did not change the expression of p-AKT significantly, but obviously decreased the expression level of p-ERK (Fig. 6G, H). Suggesting that the activated SDF-1/CXCR4 axis might further activate the ERK signaling pathway to promote leukemia cell proliferation. To confirm this conjecture, the co-culture system was incorporated with AMD3100, a specific CXCR4 suppressor, and it was found that AMD3100 could effectively reduce the p-ERK level in the up-regulated IFI6 group (Additional file 4: Figure S4). Further, the AMD3100 incorporation into the co-culture system was found capable of weakening the pro-proliferative effect of up-regulated IFI6 (Fig. 6I, J). Also, the addition of the ERK inhibitor PD98059 to the co-culture system of MSCs with up-regulated IFI6 also showed consistent results (Fig. 6K, L). As implied by these findings, the pro-proliferative function of IFI6 for leukemia cells might be exerted through the SDF-1/CXCR4/ERK signal stimulation.

### Overexpression of IFI6 in MSCs affected the gene expression profile of leukemia cells in the leukemia niche

**Fig. 7:**
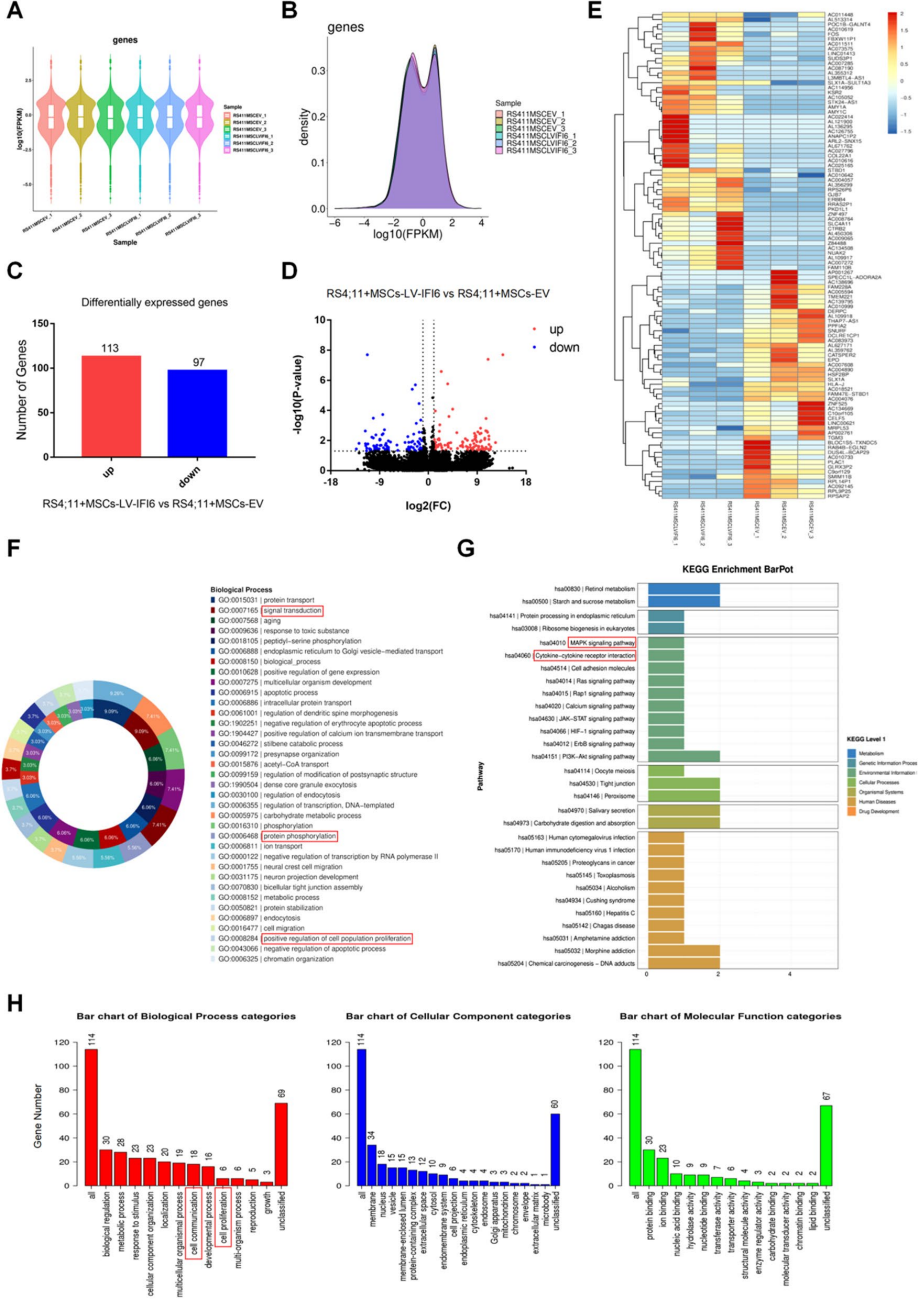
The effect of overexpression of IFI6 in MSCs on the gene expression profile of leukemia cells.** A** The distributions of gene expression in RS4;11 cells co-cultured with MSCs-EV and MSCs-LV-IFI6 for 72 h based on log_10_(FPKM), (n = 3). **B** The gene expression density of RS4;11 cells co-cultured with MSCs-EV and MSCs-LV-IFI6. **C** Histogram of differential expressed genes in RS4;11 cells co-cultured with MSCs-LV-IFI6 compared to RS4;11 cells co-cultured with MSCs-EV group based on -log_10_ (P value). **D** and **E** The volcano plots and heatmap (Top 100) of differential gene expression in RS4;11 cells co-cultured with MSCs-LV-IFI6 compared to RS4;11 cells co-cultured with MSCs-EV group based on -log_10_ (P value). **F** and **G** GO Enrichment doughnut-Biological Process (**F**) and KEGG Enrichment BarPlot (**G**) of differentially expressed genes in RS4;11 cells co-cultured with MSCs-LV-IFI6 compared to RS4;11 cells co-cultured with MSCs-EV. **H** Over-Representation Analysis (ORA) for the GO Slim summary of differentially expressed genes in RS4;11 cells co-cultured with MSCs-LV-IFI6 compared to RS4;11 cells co-cultured with MSCs-EV (Of the 210 differential genes included, 209 user IDs were identified. Among them, 114 user IDs were unambiguously mapped to 114 unique entrezgene IDs. And the GO Slim summary were based upon the 114 unique entrezgene IDs)

To further unravel IFI6's role in the molecular biological characteristics of leukemia cells, we co-cultured MSCs from EV group and LV-IFI6 group with RS4;11 cells for 72 h, respectively, and then collected leukemia cells for transcriptome sequencing analysis. Figure 7A, B displayed the distributions of gene expression and the gene expression densities of the tested samples. Through differential gene expression analysis (|log_2_FC|> 1, P < 0.05), the total number of DEGs was found to be 210 in the LV-IFI6 group, of which 113 were up-regulated and 97 were down-regulated (Fig. 7C). Figures 7D, E showed volcano and cluster plots (Top 100) of DEGs between the two groups. Through GO analysis, it was found that the biological functions enriched by differential genes included signal transduction, protein phosphorylation and positive regulation of cell population proliferation et al. (Fig. 7F). KEGG analysis revealed that the enriched signaling pathways included changes in MAPK and cytokine-cytokine receptor signaling pathway (Fig. 7G). Further, Over-Representation Analysis (ORA) (http://webgestalt.org/) showed that the enriched biological functions included cell conmmunication and cell proliferation (Fig. 7H). These data seemed to be consistent with the above experimental results in this study. Taken together, we speculated that the IFI6 level elevation in the co-cultured MSCs produced a certain effect on the gene expression profile and proliferation of leukemia cells.

## Discussion

For leukemia cells, a vital site for their survival and multiplication is the bone marrow microenvironment. TME has been reported to function crucially in the occurrence and development of leukemia [19–21]. BM-MSCs, as a pivotal microenvironmental constituent of bone marrow, are the main factor promoting leukemia progression in the TME [22, 23]. However, it remains unclear what changes have taken place in MSCs in the B-ALL niches. Through establishment of the leukemia microenvironment in vitro, this study found that MSCs in leukemia niche had undergone changes in several aspects.

Regarding the stemness markers of MSCs, Zhang et al. [24] revealed that the stemness of leukemia cell-derived MSCs did not change significantly compared with donor-derived MSCs. Nevertheless, whether MSCs are altered in leukemic niches is still unknown. In the present work, the difference of the stemness between MSCs in the leukemic niche and MSCs in mono-culture was not significant. Regarding the self-renewal traits of MSCs, Vanegas et al. [14] reported that MSCs in the co-culture system of MSCs and REH cells showed increased expression. But in our study, these indicators just presented upward trends, without revealing statistical differences. In terms of the multi-directional differentiation potential of MSCs, Zhao et al. [25] showed that MSCs derived from ALL were similar to normal MSCs, while Vicente et al. [26] suggested that ALL-MSCs have increased adipogenic capacity compared to normal MSCs. In our current work, MSCs in the leukemia niche had attenuated osteogenic and adipogenic differentiation abilities, and displayed varying degrees of senescence changes, showing agreement with prior studies by Bonilla et al. [13], Yang et al. [27] and Vanegas et al. [14]. Yuan et al. [28] also showed that after co-culture of MSCs with T-ALL cell-derived extracellular vesicles, the MSCs exhibited suppressed differentiation towards osteogenesis. In terms of apoptosis and cell cycle, we just observed changes in the MSC–Nalm-6 co-culture system. Bonilla et al. [13] found that the cell cycle of MSCs in the MSCs-REH cells co-culture system showed stagnation in G2/M phase. These results suggested that the leukemia niches constructed by different cell lines might have inconsistent experimental results, but the MSCs in the leukemia niches indeed have some changes in several aspects.

For the gene expression profiles, the current study found that MSCs in leukemia niches had significant expression changes. In a previous study, the data also showed different gene expression profiling in MSCs co-cultured with primary BCP-ALL cells; besides, survival benefit was observed in leukemia cells after co-culture with MSCs [29]. This finding is consistent with ours. As for the possible biological functions of MSCs, studies have shown that MSCs could promote leukemia progression [10, 18]. In the present work, we found that the differential genes of MSCs in leukemia niche were enriched to include several biological functions that promote tumor progression, which suggested that MSCs might be critical to the persistence and deterioration of leukemia cells.

To further describe how DEGs in MSCs affect the leukemia cells, in this study, we screened IFI6, an interferon-stimulated gene, which though has not been explored in B-ALL. During the occurrence and development of viral infectious diseases [30, 31], autoimmune diseases [32, 33] and some tumors [16, 34–37], IFI6 is often highly expressed, which exerts the functions of resisting apoptosis and viruses, as well as promoting tumor progression. Liu et al. [17] found that IFI6 was increased in patients with esophageal squamous cell carcinoma, the overexpression of IFI6 was closely related to the invasive phenotype and poor outcome. In an ovarian cancer research, the overexpression of IFI6 could facilitate the multiplication of tumor cells and mediate their chemoresistance [15]. In addition, IFI6 is regarded as a crucial predictor of poor outcome in breast cancer [34, 38]. In the study of hematological tumors, aberrantly expressed IFI6 in multiple myeloma is an important factor leading to the chemoresistance of myeloma cells [39]. In the present work, we found that IFI6 might be an important component in the B-ALL microenvironment that promotes the proliferation of leukemia cells.

Regarding the tumor progression-promoting mechanism of IFI6 at the molecular level, Cheriyath et al. [39] found that IFI6 regulated the balance between Bcl-2 and Bim expression to resist apoptosis. And Liu et al. [17] revealed that the mitochondrial Ca^2+^ overload could be induced by down-regulation of IFI6 to induce tumor cells apoptosis. In this study, we explored and found that highly expressed IFI6 in MSCs promoted the activation of the SDF-1/CXCR4 axis initiation in the TME, which served as a mediator in the stromal component–tumor cell interaction [40, 41]. Multiple studies have attempted to make leukemia cells more sensitive to chemotherapeutics by disrupting their interaction using the CXCR4 inhibitor AMD3100 [42–44]. In this study, AMD3100 also effectively attenuate the pro-proliferative effect of IFI6 on leukemia cells. AKT and ERK signaling pathways are key pathways that promote tumor progression in leukemia [45, 46]. In the present work, IFI6 was found capable of initiating the ERK signaling pathway via the SDF-1/CXCR4 axis, thereby facilitating the leukemia cell multiplication. Suggesting that targeting ERK pathway in leukemia niches is probably a valid strategic option for reducing the leukemia cell multiplication.

Finally, we also found in this work that the increased expression of IFI6 in MSCs had some effects on the gene expression profile and biological functions of leukemia cells through RNA sequencing. Although this has not been reported in other studies, due to the small number of differential genes enriched in the GO/KEGG entries of our interest in this dataset and the small differences between the two groups, further study is needed. In addition, although this study interestingly found that increased expression of IFI6 in MSCs might be a key factor leading to the proliferation of B-ALL cells through in vitro and in vivo experiments, the current exploration is preliminary and limited to the B-ALL cell lines, more in-depth studies are needed to demonstrate the role of IFI6 in ALL.

## Conclusion

**Fig. 8:**
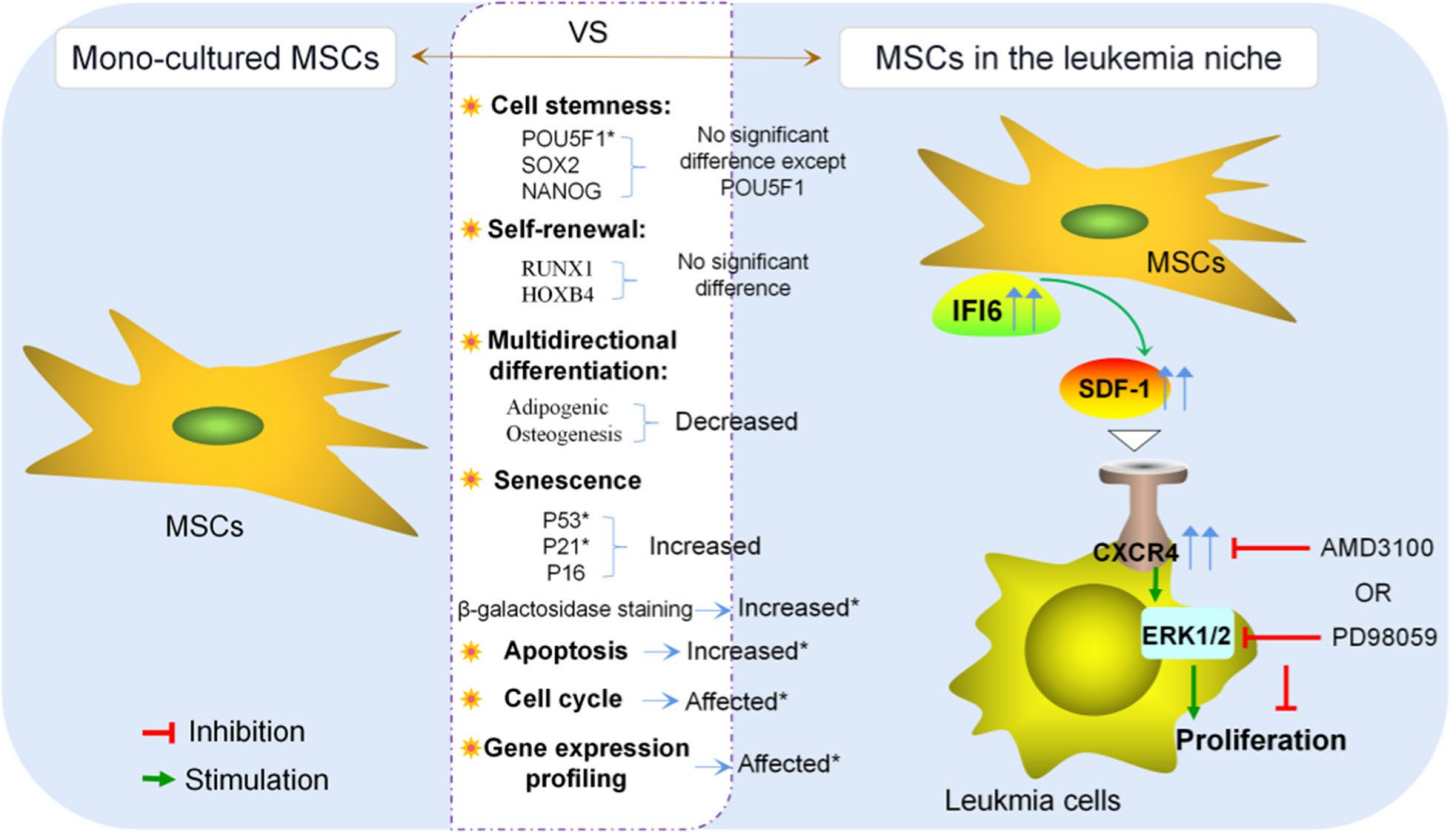
Mechanism diagram. MSCs in leukemia niche exhibite alterations in multilineage differentiation, cell cycle, cell senescence and gene expression profiles, and exert pro-proliferative effects through overexpression of IFI6. Mechanistically, IFI6 might promote the proliferation of B-ALL cells by stimulating the SDF-1/CXCR4 axis to activate the ERK signaling pathway, targeting IFI6 or related signaling pathways might be an important measure to reduce leukemia cell proliferation. *The indicator is statistically different in at least one of the comparisons

Taken together, our results demonstrated that MSCs in leukemia niches exhibit varying degrees of alterations. And the high expression of IFI6 in leukemia niche is probably critical to the leukemia proliferation promotion by MSCs, targeting IFI6 or related signaling pathways might be an important measure to reduce leukemia cell proliferation (Fig. 8).

### Supplementary Information

**Additional file 1: Figure S1**. The cell transfection ratio of IFI6 was observed by fluorescence microscopy. **A** The cell transfection ratio of IFI6 in MSCs-EV and MSCs-LV-IFI6 group under fluorescence microscopy (100 ×). **B** The cell transfection ratio of IFI6 in MSCs-EV and MSCs-Si-IFI6 group under fluorescence microscopy (100 ×).

**Additional file 2: Figure S2**. Down-regulation of IFI6 in MSCs could reduce the proliferation of leukemia cells. **A** and **B** The mRNA and protein levels of IFI6 in MSCs with CON, EV and Si-IFI6, mean ± SD, n = 3. **C** The numbers of cell proliferation of Nalm-6 and RS4;11 cells co-cultured with blank, MSCs, MSCs-EV and MSCs-Si-IFI6 groups after 24 h, 48 h, 72 h and 96 h of cells incubation, mean ± SEM, n = 3, the “*” in the figure represents a significant difference between the MSCs-EV and MSCs-Si-IFI6 groups. *P < 0.05, **P < 0.01, ***P < 0.001.

**Additional file 3: Figure S3**. IFI6 promotes the growth and proliferation of Nalm-6 cells in vivo. **A** Schematic diagram of subcutaneous tumor formation in mice. **B** The size of subcutaneous tumors in the Nalm-6 injection group, Nalm-6 + MSCs injection group, Nalm-6 + MSCs-EV injection group, and Nalm-6 + MSCs-LV-IFI6 injection group, n = 3. **C** Average tumor weight in each group was calculated at 34 days after injection. mean ± SD. *P < 0.05, **P < 0.01. **D** Average tumor volume in each group was evaluated at 34 days after injection. mean ± SD. **P < 0.01.

**Additional file 4: Figure S4**. AMD3100 could reduce the expression level of p-ERK in the up-regulated IFI6 group. **A** and **B** The expression levels of p-ERK in Nalm-6/RS4;11 co-cultured with MSCs-CON, MSCs-EV, MSCs-LV-IFI6 and MSCs-LV-IFI6 + AMD3100 (20 μM) for 72 h by western blot, mean ± SD, n = 3. *P < 0.05, **P < 0.01, ***P < 0.001.

## Data Availability

Gene expression profiles generated in this paper have been deposited in the GEO under accession number: GSE212209, GSE213038. All other vectors described in the present study are available from the authors upon request.

## Abbreviations

ADIPOQ: Adiponectin, C1Q and collagen domain containing
B-ALL: B-cell acute lymphoblastic leukemia
BCP-ALL: B-Cell precursor acute lymphoblastic leukemia
BGLAP: Bone gamma-carboxyglutamate protein
BM-MSCs: Bone marrow mesenchymal stem cells
CXCR4: Chemokine receptor 4
DAVID: Database for annotation visualization and integrated discovery
DEGs: Differentially expressed genes
FDR: Flase discovery rate
GEO: Gene expression omnibus
GO: Gene ontology
GSEA: Gene set enrichment analysis
HOXB4: Homeobox B4
IFI44L: Interferon induced protein 44 like
IFI6: Interferon alpha inducible protein 6
IFIT1: Interferon induced protein with tetratricopeptide repeats 1
IFIT3: Interferon induced protein with tetratricopeptide repeats 3
IFITM1: Interferon induced transmembrane protein 1
IHC: Immunohistochemistry
ISG15: ISG15 ubiquitin like modifier
KEGG: Kyoto encyclopedia of genes and genomes
MX1: MX dynamin like GTPase 1
NANOG: Nanog homeobox
NOD/SCID: Nonobese diabetes/severe combined immunodeficiency
ORA: Over-representation analysis
PBS: Phosphate buffer solution
POU5F1: POU class 5 homeobox 1
PPAR-γ: Peroxisome proliferator activated receptor γ
qRT-PCR: Quantitative real-time PCR
RIPA: Radio immunoprecipitationassay
RUNX1: RUNX family transcription factor 1
RUNX2: RUNX family transcription factor 2
SDF-1: Stromal cell derived factor 1
SOX2: SRY-box transcription factor 2
TME: Tumor microenvironment
