# Supplementary figures and images for: BM-MSCs display altered gene expression profiles in B-cell acute lymphoblastic leukemia niches and exert pro-proliferative effects via overexpression of IFI6
Source: J Transl Med. 2023 Sep 5;21:593. doi: 10.1186/s12967-023-04464-1 (PMC10478283; doi:10.1186/s12967-023-04464-1)

**A****White light****Brightfield****Merge****EV**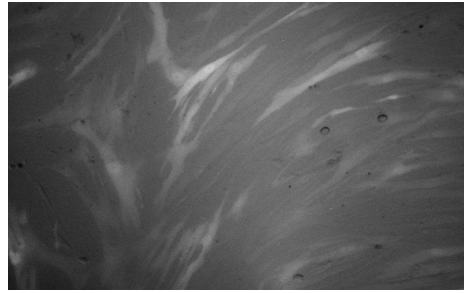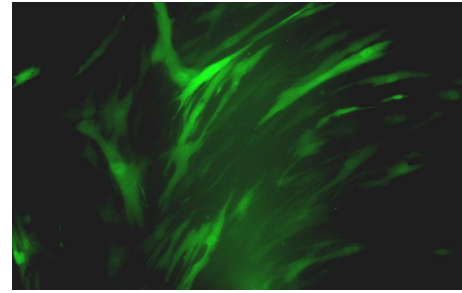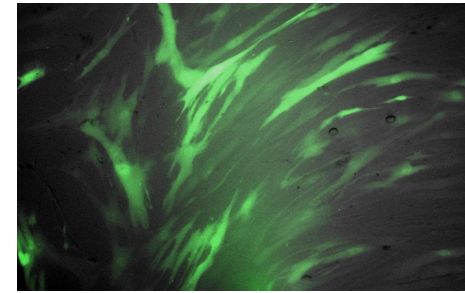**LV-IFI6**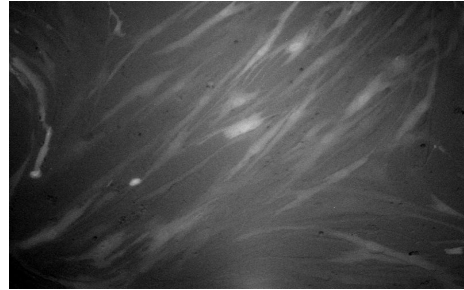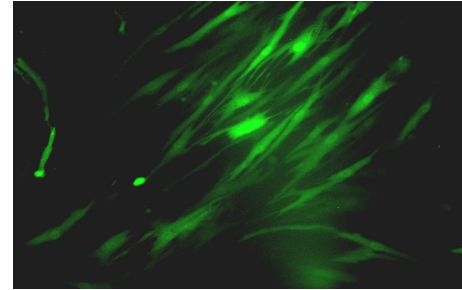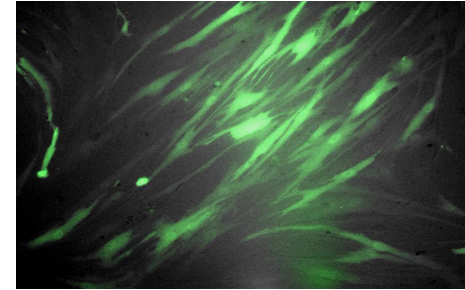**B****White light****Brightfield****Merge****EV**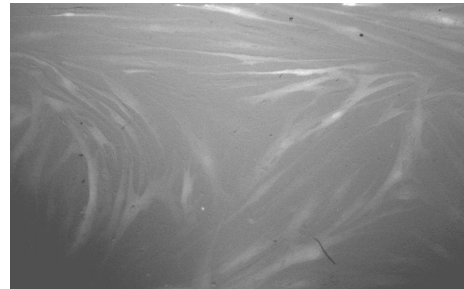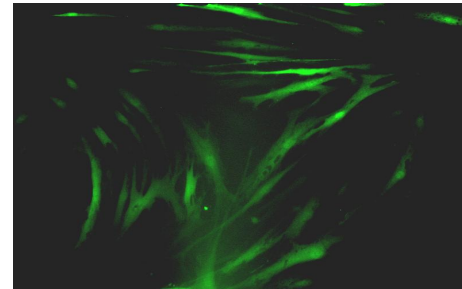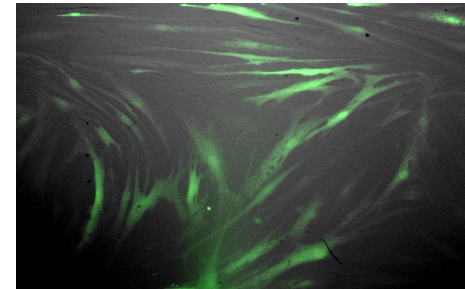**Si-IFI6**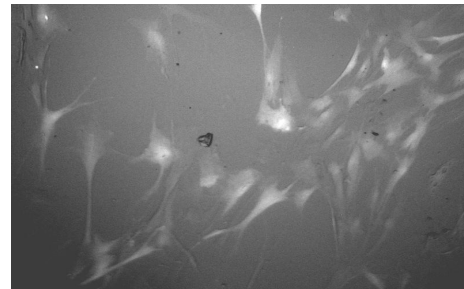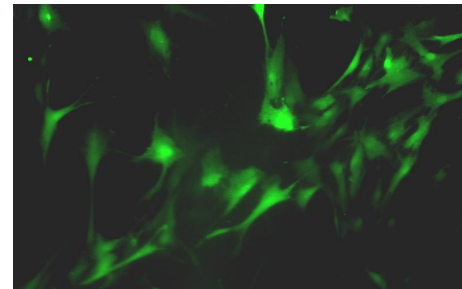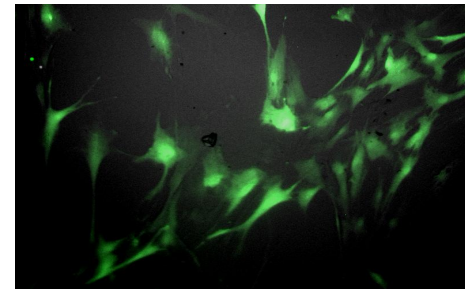

Supplement: Supplementary file 1 — Additional file 1: Figure S1. The cell transfection ratio of IFI6 was observed by fluorescence microscopy. A The cell transfection ratio of IFI6 in MSCs-EV and MSCs-LV-IFI6 group under fluorescence microscopy (100 ×). B The cell transfection ratio of IFI6 in MSCs-EV and MSCs-Si-IFI6 group under fluorescence microscopy (100 ×). [file 12967_2023_4464_MOESM1_ESM.pdf]

**A**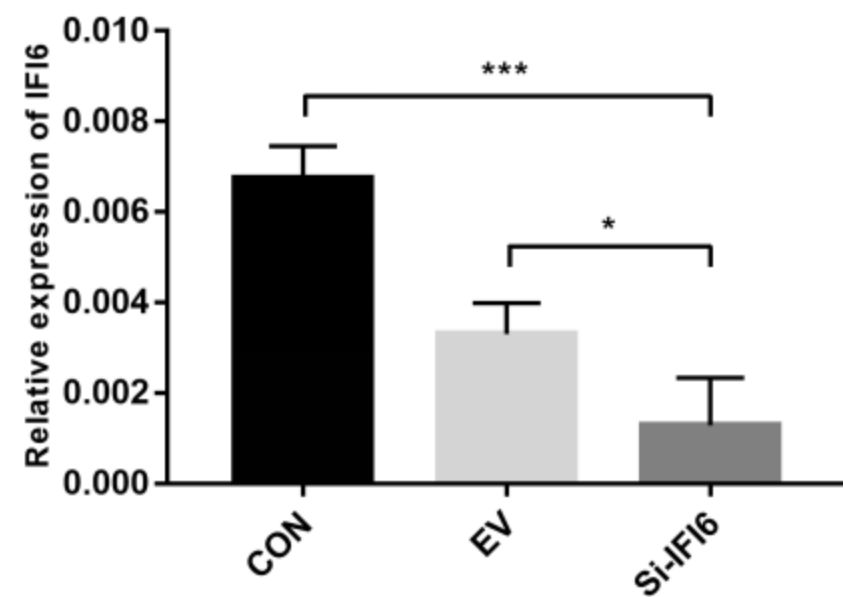**B**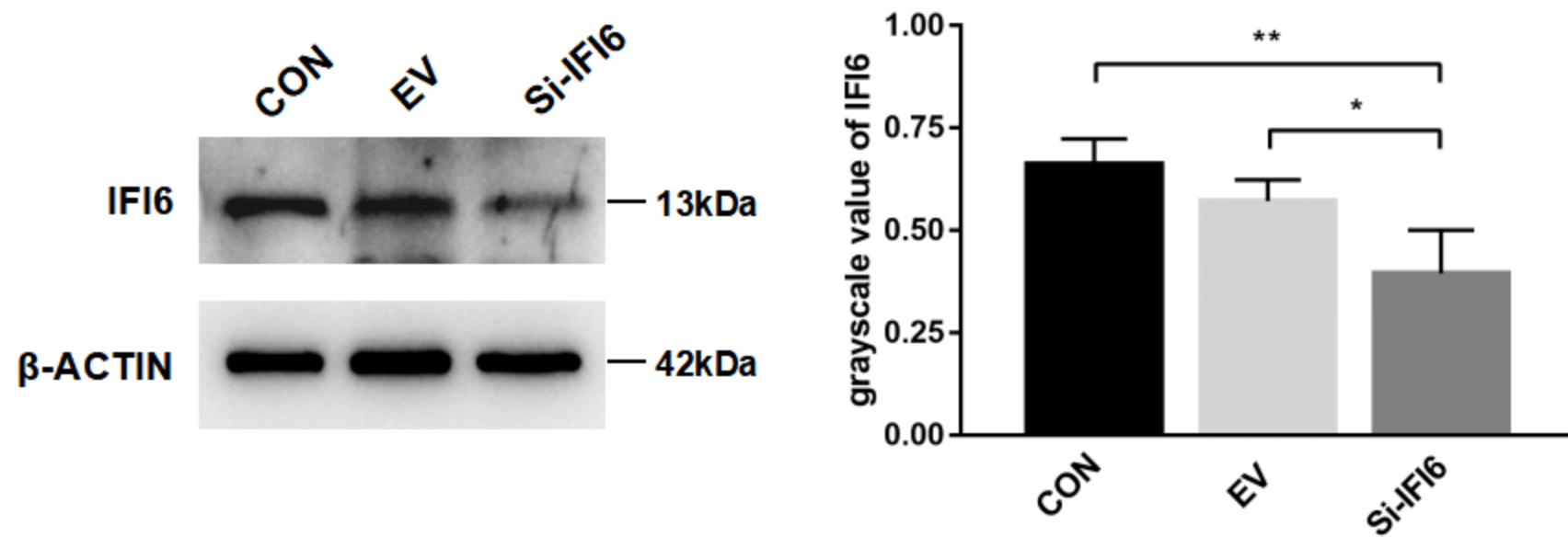**C**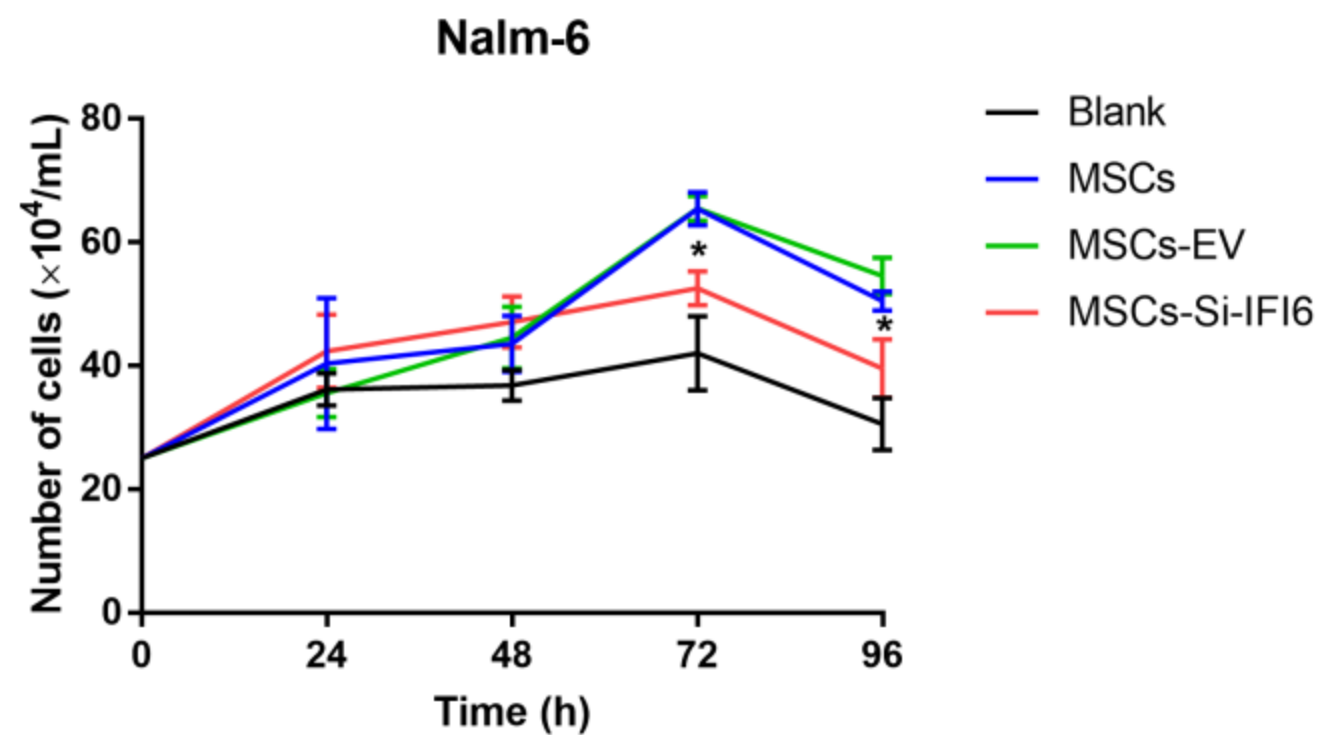**D**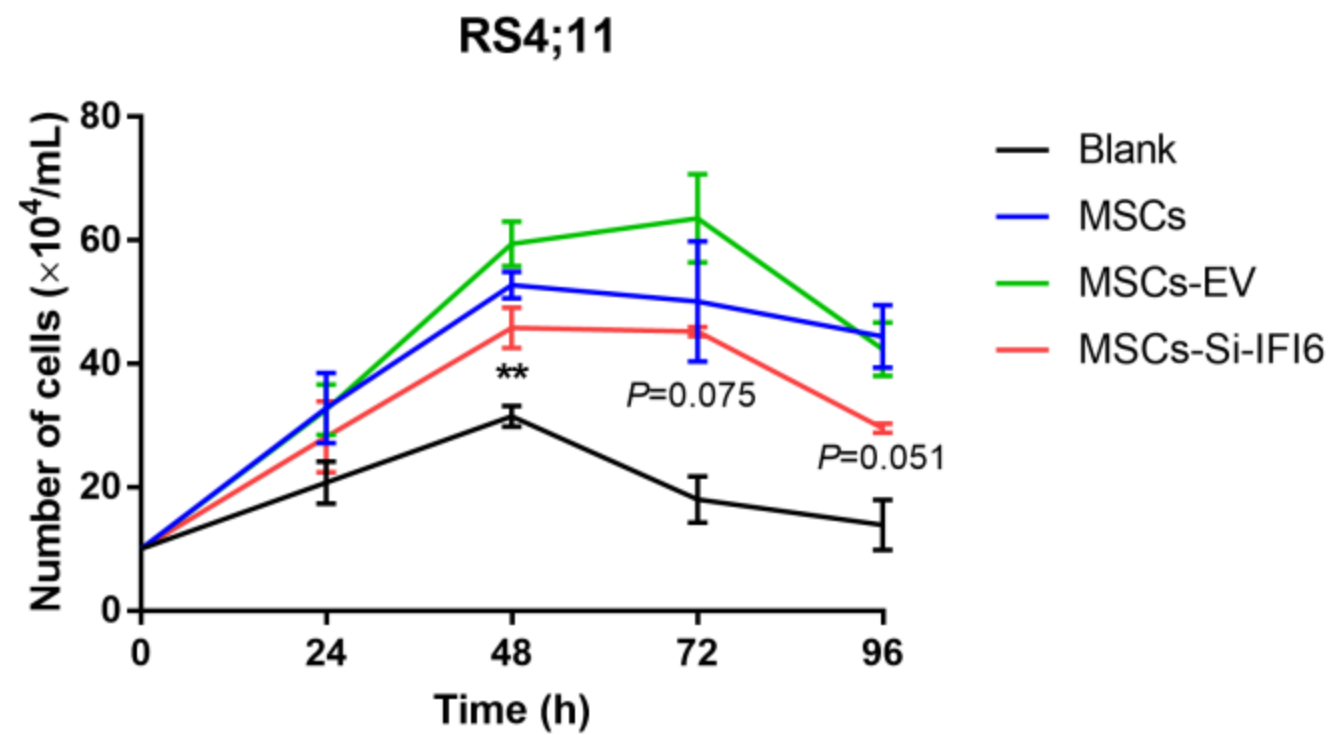

Supplement: Supplementary file 2 — Additional file 2: Figure S2. Down-regulation of IFI6 in MSCs could reduce the proliferation of leukemia cells. A and B The mRNA and protein levels of IFI6 in MSCs with CON, EV and Si-IFI6, mean ± SD, n = 3. C The numbers of cell proliferation of Nalm-6 and RS4;11 cells co-cultured with blank, MSCs, MSCs-EV and MSCs-Si-IFI6 groups after 24 h, 48 h, 72 h and 96 h of cells incubation, mean ± SEM, n = 3, the “*” in the figure represents a significant difference between the MSCs-EV and MSCs-Si-IFI6 groups. *P < 0.05, **P < 0.01, ***P < 0.001. [file 12967_2023_4464_MOESM2_ESM.pdf]

**A**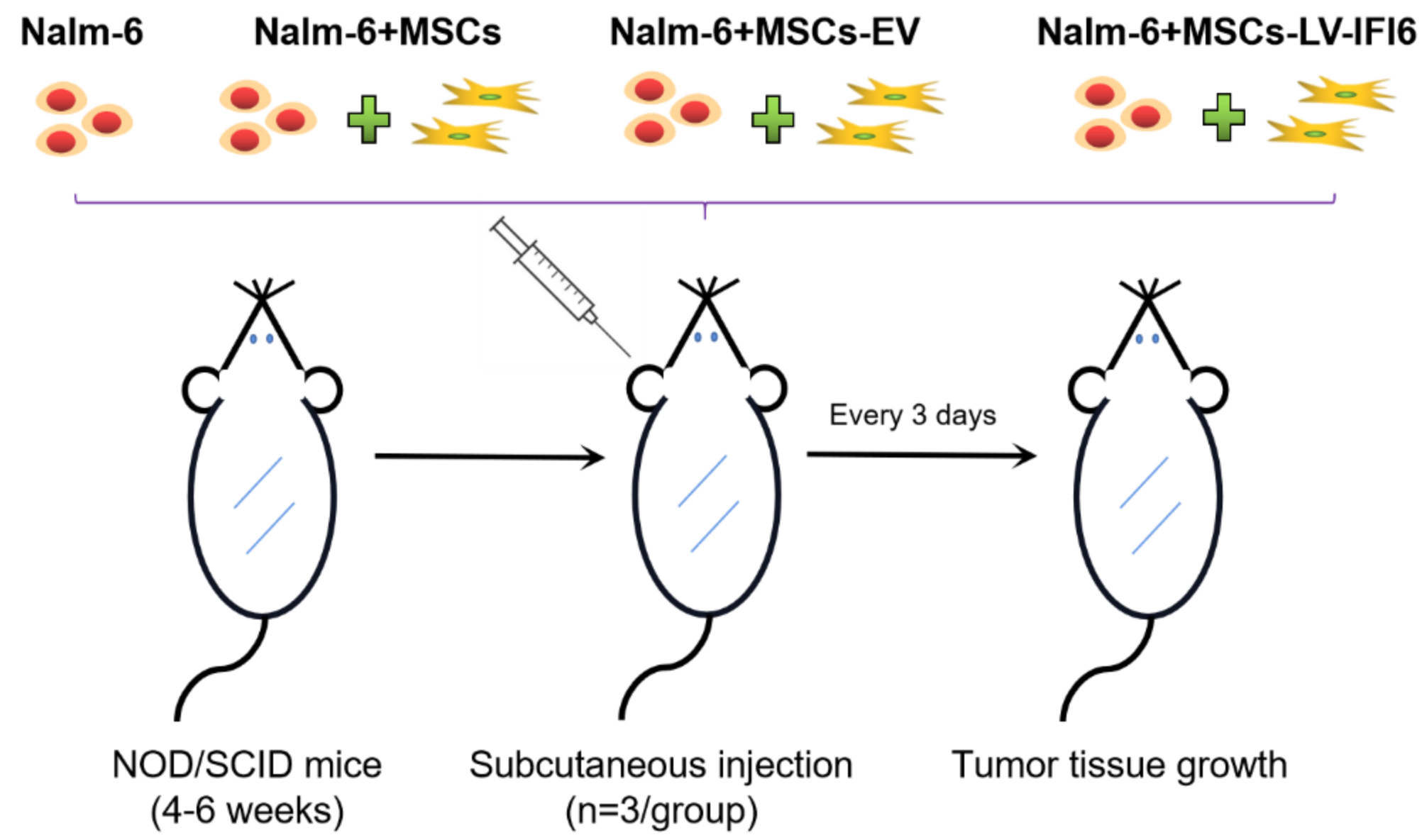**B**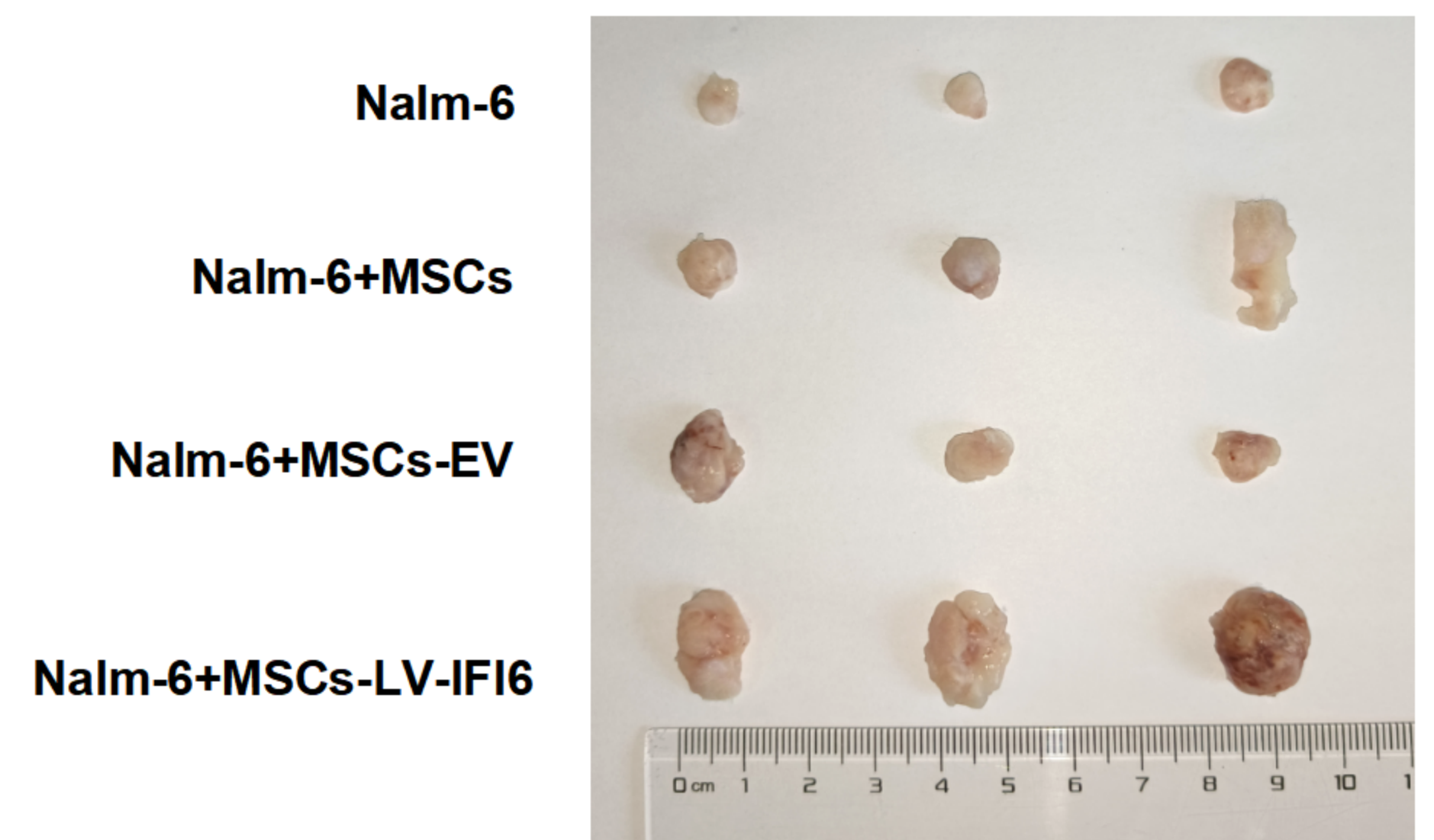**C**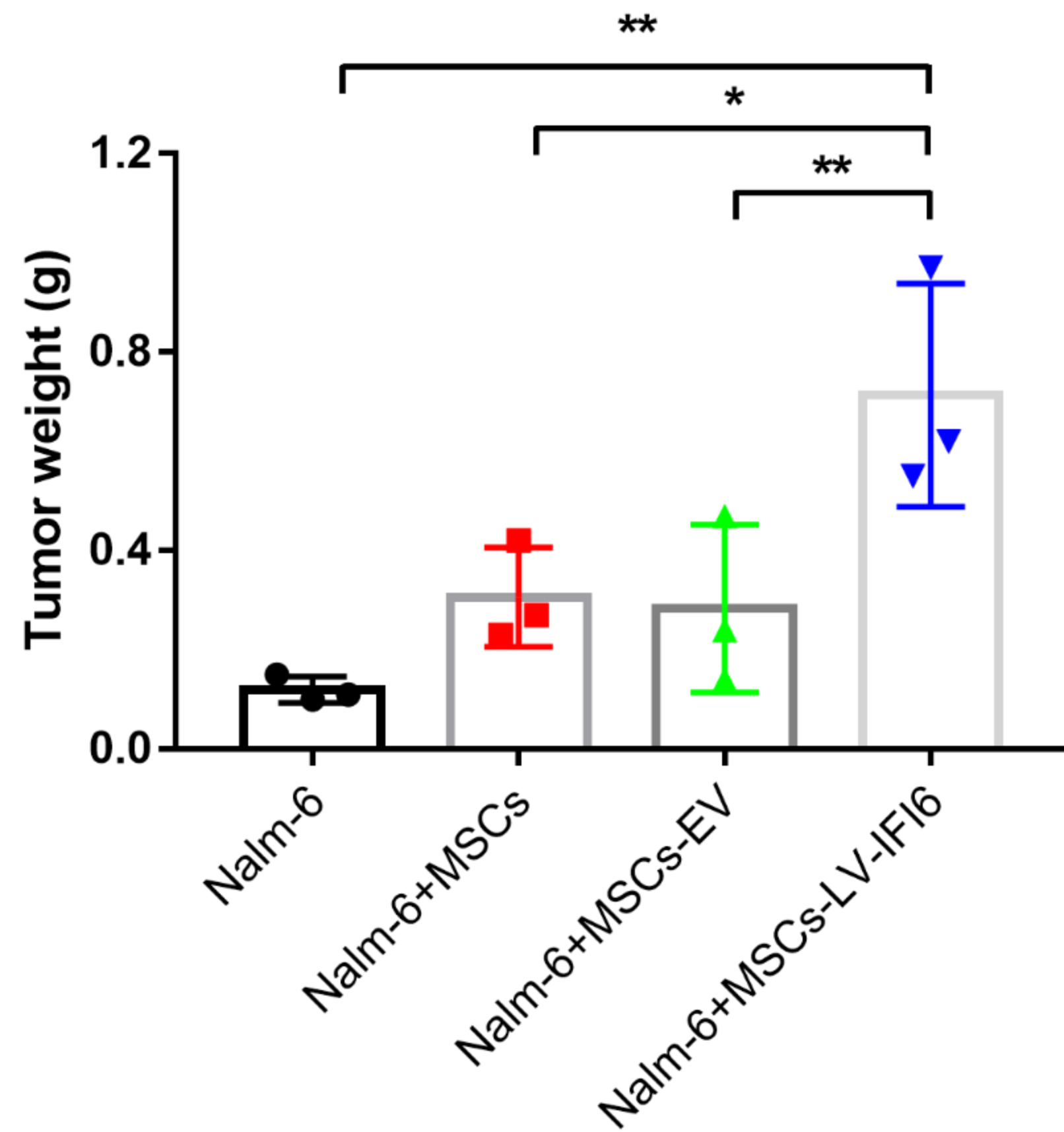**D**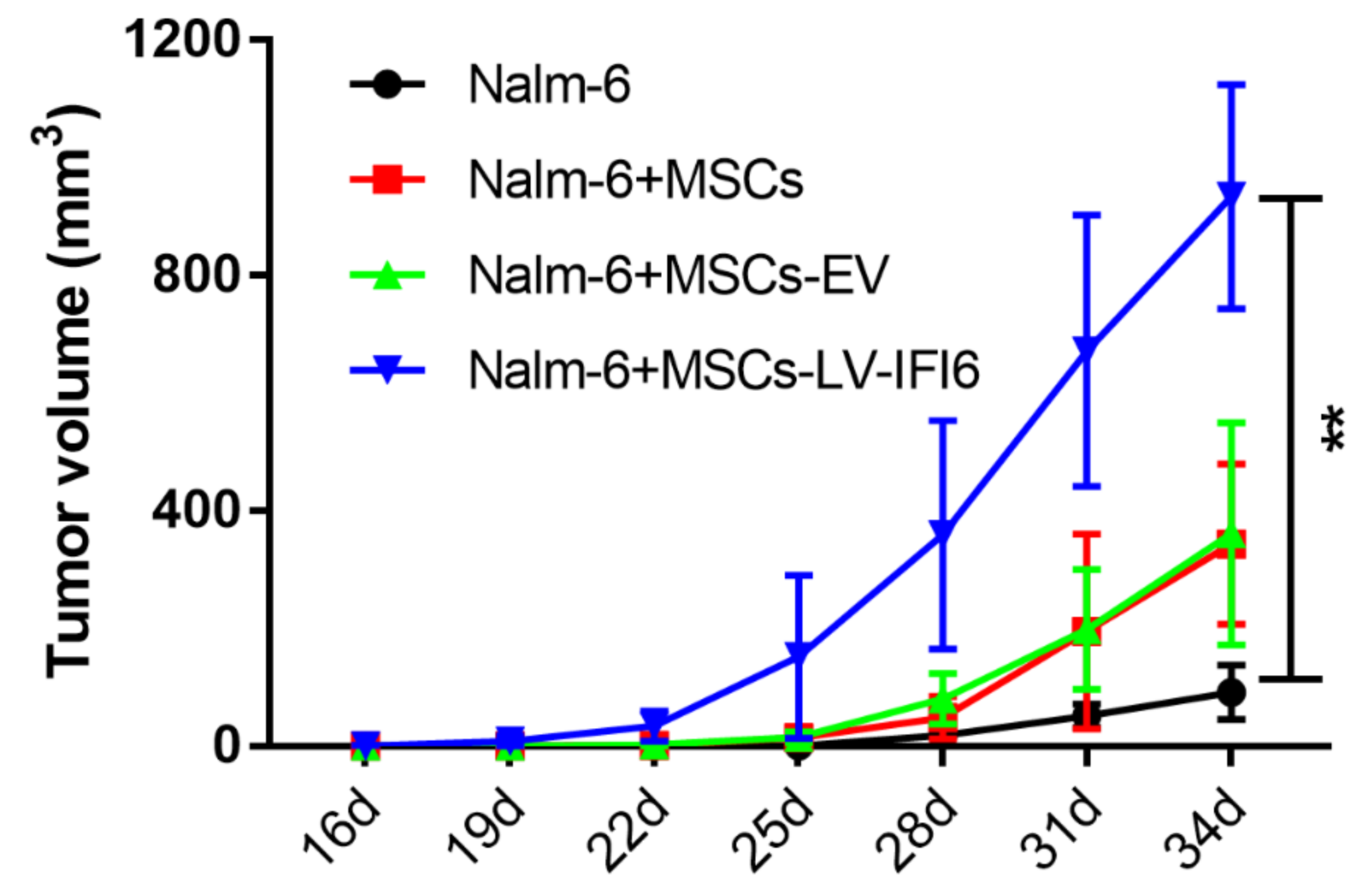

Supplement: Supplementary file 3 — Additional file 3: Figure S3. IFI6 promotes the growth and proliferation of Nalm-6 cells in vivo. A Schematic diagram of subcutaneous tumor formation in mice. B The size of subcutaneous tumors in the Nalm-6 injection group, Nalm-6 + MSCs injection group, Nalm-6 + MSCs-EV injection group, and Nalm-6 + MSCs-LV-IFI6 injection group, n = 3. C Average tumor weight in each group was calculated at 34 days after injection. mean ± SD. *P < 0.05, **P < 0.01. D Average tumor volume in each group was evaluated at 34 days after injection. mean ± SD. **P < 0.01. [file 12967_2023_4464_MOESM3_ESM.pdf]

**A****Nalm-6**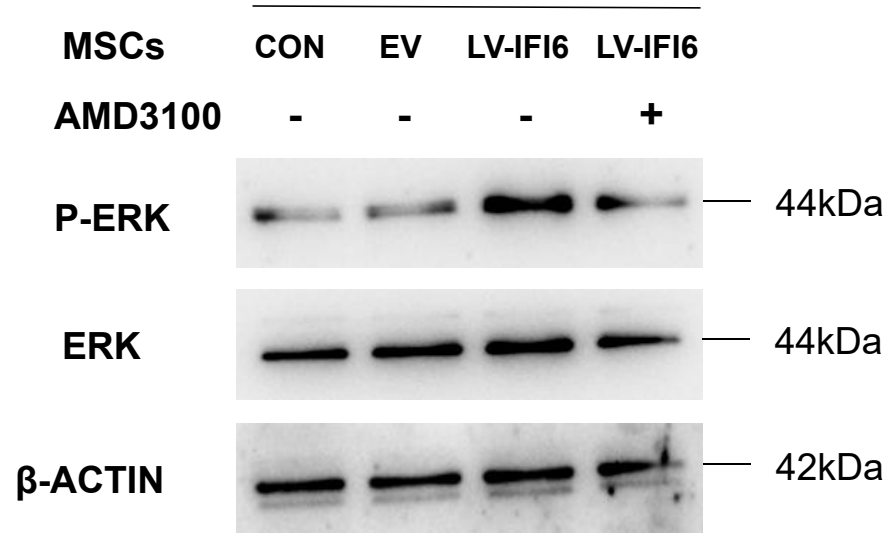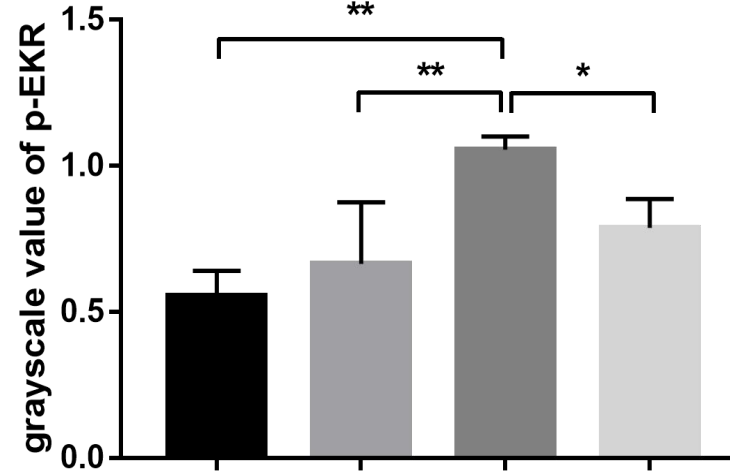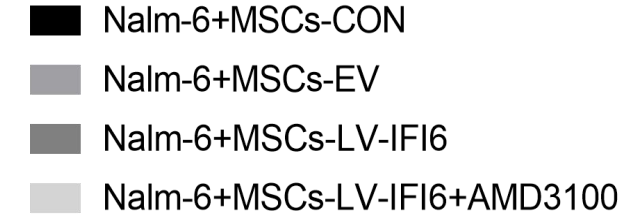**B****RS4;11**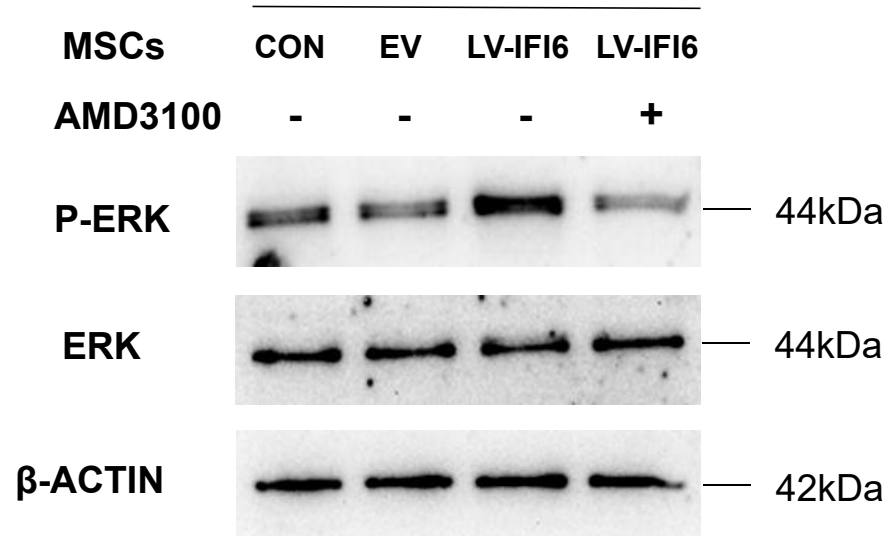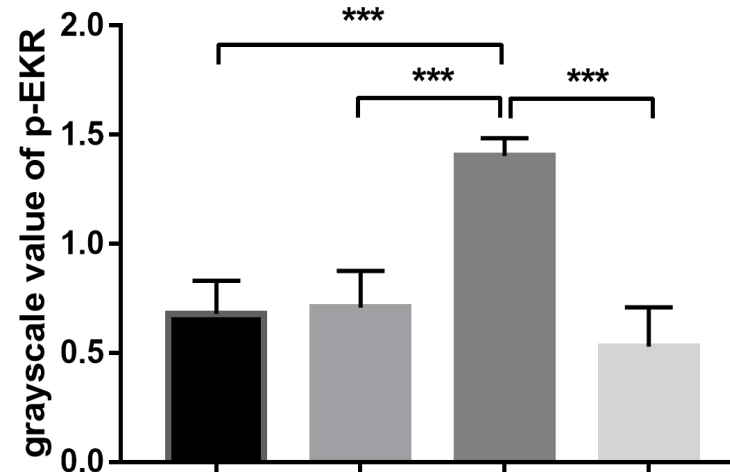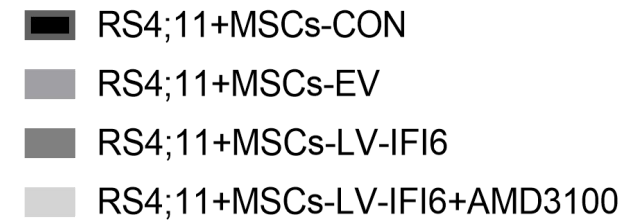

Supplement: Supplementary file 4 — Additional file 4: Figure S4. AMD3100 could reduce the expression level of p-ERK in the up-regulated IFI6 group. A and B The expression levels of p-ERK in Nalm-6/RS4;11 co-cultured with MSCs-CON, MSCs-EV, MSCs-LV-IFI6 and MSCs-LV-IFI6 + AMD3100 (20 μM) for 72 h by western blot, mean ± SD, n = 3. *P < 0.05, **P < 0.01, ***P < 0.001. [file 12967_2023_4464_MOESM4_ESM.pdf]
